# Probing the Effect of Photovoltaic Material on V_oc_ in Ternary Polymer Solar Cells with Non-Fullerene Acceptors by Machine Learning

**DOI:** 10.3390/polym15132954

**Published:** 2023-07-05

**Authors:** Di Huang, Zhennan Li, Kuo Wang, Haixin Zhou, Xiaojie Zhao, Xinyu Peng, Rui Zhang, Jipeng Wu, Jiaojiao Liang, Ling Zhao

**Affiliations:** 1 College of Railway Transportation, Hunan University of Technology, Zhuzhou 412008, China; dihuang@hut.edu.cn (D.H.); m21085400035@stu.hut.edu.cn (Z.L.); king_20211225@163.com (K.W.); m22085400062@stu.hut.edu.cn (H.Z.); zxj15074980122@163.com (X.Z.); zhang_030720@163.com (R.Z.); jipengwu243@163.com (J.W.); 2 College of Electrical and Information Engineering, Hunan University of Technology, Zhuzhou 412008, China; m22085800061@hut.edu.cn; 3 Qinghai Provincial Key Laboratory of Nanomaterials and Nanotechnology, Qinghai Minzu University, Qinghai 810007, China; 4 Shandong Provinical Key Laboratory of Optical Communication Science and Technology, School of Physical Science and Information Technology, Liaocheng University, Liaocheng 252059, China

**Keywords:** polymer solar cells, open circuit voltage, machine learning, molecular descriptors, molecular fingerprints

## Abstract

The power conversion efficiency (PCE) of ternary polymer solar cells (PSCs) with non-fullerene has a phenomenal increase in recent years. However, improving the open circuit voltage (V_oc_) of ternary PSCs with non-fullerene still remains a challenge. Therefore, in this work, machine learning (ML) algorithms are employed, including eXtreme gradient boosting, K-nearest neighbor and random forest, to quantitatively analyze the impact mechanism of V_oc_ in ternary PSCs with the double acceptors from the two aspects of photovoltaic materials. In one aspect of photovoltaic materials, the doping concentration has the greatest impact on V_oc_ in ternary PSCs. Furthermore, the addition of the third component affects the energy offset between the donor and acceptor for increasing V_oc_ in ternary PSCs. More importantly, to obtain the maximum V_oc_ in ternary PSCs with the double acceptors, the HOMO and LUMO energy levels of the third component should be around (−5.7 ± 0.1) eV and (−3.6 ± 0.1) eV, respectively. In the other aspect of molecular descriptors and molecular fingerprints in the third component of ternary PSCs with the double acceptors, the hydrogen bond strength and aromatic ring structure of the third component have high impact on the V_oc_ of ternary PSCs. In partial dependence plot, it is clear that when the number of methyl groups is four and the number of carbonyl groups is two in the third component of acceptor, the V_oc_ of ternary PSCs with the double acceptors can be maximized. All of these findings provide valuable insights into the development of materials with high V_oc_ in ternary PSCs for saving time and cost.

## 1. Introduction

In recent years, polymer solar cells (PSCs) have become a hot spot in the photovoltaic field for integration with buildings due to their superior flexibility, light weight and high optical stability [1,2,3,4]. At present, the power conversion efficiency (PCE) of PSCs has exceeded 18% [5], which is mainly due to synthesizing new non-fullerene acceptors (NFAs), using new liquid/solid additives, developing layer-by-layer (LbL) fabrication process and applying the ternary strategy [6,7,8,9,10]. However, the V_oc_ in the ternary PSCs is still lower than that of perovskite solar cells [11]. Some methods have been delivered, including effectively adjusting the NFA energy levels, to form the good energy level offset with the donor material for improving the V_oc_ in devices [12,13]. Yong Cui et al. incorporated F-BTA3 as the third component in the solar cell with PBQx-TF:eC9-2Cl to form the cascaded energy level, which had a significant increase of V_oc_ from 0.86 V to 0.879 V, meanwhile achieving a maximum PCE of 19.0% in ternary PSC [14]. Junyan Yang et al. introduced the small molecule donor of BTID-2F into PM6:Y6 to obtain lower energy loss, resulting in a V_oc_ increase from 0.84 V of binary PSCs to 0.85 V of ternary PSCs, and PCE increased from 16.62% to 17.98%, which is attributed to the suppressed recombination and morphological optimization [15]. Guanshui Xie’s team also reported that adding the polymer donor of P1, with a deeper highest occupied molecular orbital (HOMO) energy level into the PM6:Y6 for reducing the HOMO level offset between Y6 and P1, which helped to decrease the energy loss and thus increase the V_oc_ in PSCs [16]. These examples illustrate that adding the third component can effectively improve the V_oc_ and PCE of ternary PSCs. However, the current research is basically based on the qualitative relationship studies in specific material system, and the quantitative analysis of the influence of the electronic properties and chemical structure of materials on PSCs’ V_oc_ remains to be further studied.

Currently, density functional theory calculation, molecular dynamics simulation, multi-scale simulation model of semiconductor devices and empirical formula derivation have been applied for investigating the quantitative influence on PSC performance [17,18,19]. These research methods have important guidance for material screening and device optimization in solar cells. However, they often consume a lot of time and financial cost. Machine learning (ML) allows researchers to excavate the hidden physical laws behind big data and draw reliable conclusions from the perspective of algorithms for more effectively accelerating material design and performance optimization in PSCs to save time and money [20,21,22]. Currently, ML has been widely applied in the field of PSCs [23,24,25,26,27]. Kakaraparthi Kranthiraja et al. developed a series of novel π-conjugated polymer donor materials for NFAs based on the random forest (RF) model [23]. Sijing Zhong et al. reviewed the key challenges of ML in the design and improvement of PCE in photovoltaic materials in recent years and gave the guiding suggestions for reaching the high performance in devices [24]. Jinliang Wang et al. used ML to screen out 15 acceptors to match PTB7-Th for gaining more than 13% PCE [17]. In addition, our group applied ML to predict the champion PCE of ternary PSCs and screen the optimal doping ratio of the third component [26]. Furthermore, we also elucidated the matching energy levels of binary PSCs materials to enhance the V_oc_ through ML [27]. Exploring organic materials and understanding hidden chemical information via ML have become a new research model. So far, the V_oc_ in many classical binary PSCs still highly depends on the band gap between the HOMO energy level of the donor and the lowest unoccupied molecular orbital (LUMO) energy level of the acceptor [28]. However, the addition of the third component makes the mapping relationship between the energy level of materials and the V_oc_ more complicated, resulting in the unclear influence mechanism of the V_oc_ in ternary PSCs. In addition, the selection of the third component for high-efficiency ternary PSCs with non-fullerene often requires trial-and-error experiments. Therefore, there is still plenty of research space for utilizing ML to reveal the intrinsic relationship among the physical and chemical properties of materials and the V_oc_.

**Figure 1:**
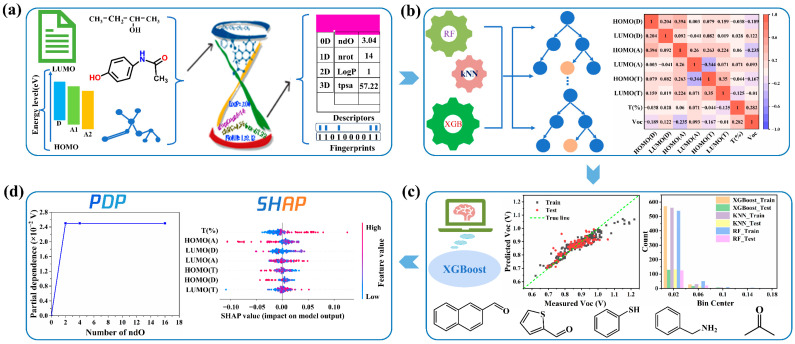
The machine learning process used in this work. (**a**) Dataset collection, (**b**) model building, (**c**) performance evaluation and (**d**) visual interpretation. Additionally, PDP stands for partial dependence plots, and SHAP represents the shapely additive explanations in the illustration.

Herein, we analyze the important impact factors of the V_oc_ in ternary PSCs with double-acceptors and explore the hidden correlation between the characteristics of non-fullerene molecules and V_oc_ in ternary PSCs with double-acceptors via frontier molecular orbitals (FMOs), molecular descriptors (MDs) and molecular fingerprints (MFs). To our best knowledge, there is no research report to investigate the effect of MDs and MFs in non-fullerene materials on V_oc_ in ternary PSCs with double-acceptors, and the overall process of ML is shown in Figure 1, which includes dataset collection, model building, performance evaluation and visual interpretation. The detail of the whole process is displayed in Materials and Methods. Moreover, Table S1 defines all of abbreviations, symbols, compounds, etc., in this work. We employ three different ML algorithms including K-nearest neighbors (KNN), RF and eXtreme gradient boosting (XGBoost) to investigate the complex relationship between FMOs of materials and V_oc_, and we also use the preferred XGBoost to study the influence of the physical and chemical properties of the third component on V_oc_. Additionally, the shapely additive explanations (SHAP) are used to analyze the influence of different input features in the XGBoost model and quantify the contribution of each input feature for V_oc_. In addition, combined with the division of the chemical structure in the third component material by MDs and MFs, the important influence of methyl and carbonyl functional groups on V_oc_ is identified. It is worth mentioning that the relative amounts of methyl and carbonyl groups are quantified through partial dependence plots (PDP). Therefore, our results provide a guideline for the preparation of ternary PSCs with high V_oc_: (1) the optimal range of the third component energy level, (2) the visual relationship between molecular fragments of NFA materials and ternary PSCs’ V_oc_ and (3) the marked key molecular functional groups for enhancing V_oc_.

## 2. Materials and Methods

### 2.1. Dataset Collection

All data are from 109 publications of non-fullerene ternary PSCs in the last five years, which are recorded as Data1 in the Supporting Information. Data1 contains seven input features (HOMO and LUMO of the donor (HOMO_(D)_ and LUMO_(D)_), HOMO and LUMO of the acceptors (HOMO_(A)_ and LUMO_(A)_), HOMO and LUMO of the third component (HOMO_(T)_ and LUMO_(T)_), the concentration of the third component (T_(%)_) and one output feature (V_oc_)) with a total of 727 sets of data points. In part 3.1 of this paper, when studying the influence FMOs of donor and acceptor on V_oc_, the FMOs and the T_(%)_ are used as input features, and V_oc_ is the output feature. In part 3.2 of this work, when researching the effects of molecular composition and molecular properties on V_oc_, the third component of Data1 is trans-coded into two new sub-datasets via the ChemDes platform, which are the two-dimensional MDs and MFs, respectively. In addition, C_V_oc_ is the V_oc_ in the binary PSCs without the third component as the extra input feature, and the output feature of V_oc_ is obtained at the value corresponding to the maximum PCE in the ternary PSCs.

### 2.2. Model Building

The ML models are built based on the Python language environment. The prediction model is established by K-nearest neighbors (KNN), random forest (RF) and eXtreme gradient boosting (XGBoost) algorithms. Among them, the KNN algorithm is a kind of non-parametric statistical lazy algorithm. For predicting the target value, the average of the K nearest values is assigned. In other words, similar inputs have similar outputs. The RF algorithm is a very representative bagging ensemble algorithm. All of the base evaluators are the decision trees. The XGBoost algorithm is built by the parallel construction of regression trees through multi-threading, which can control the model during the entire training process through a series of hyperparameters to greatly improve the speed and accuracy of model training. In addition, 20% of the total data in Table S5 is randomly selected as the testing set, while the remaining 80% is used as the training set (the training set and the testing set are mutually exclusive), and then the model is trained by using three type of ML algorithms. Random sampling ensures a good balance between the training and testing sets for preventing overfitting of the models. Then, the performance of the model is evaluated based on the predicted results. The model with the best performance results is selected for the predictive interpretation and the subsequent studies.

### 2.3. Performance Evaluation

As shown in Equations (1)–(4), the root mean square error (*RMSE*), mean absolute error (*MAE*), mean absolute percentage error (*MAPE*) and Pearson correlation coefficient (*r*) are used to evaluate the model with regression problems:

(1) $$RMSE=\sqrt{\frac{\sum_{i=1}^{n}{\left(y_{i}^{\prime }-y_{i}\right)}^{2}}{n}}$$

(2) $$MAE=\frac{1}{n}\overset{n}{\underset{i=1}{\sum }}\left|\left(y_{i}-\hat{y_{i}}\right)\right|$$

(3) $$MAPE=\frac{100\%}{n}\overset{n}{\underset{i=1}{\sum }}|\frac{{\hat{y}}_{i}-y_{i}}{y_{i}}|$$

(4) $$r=\frac{\Sigma \left(x-m_{x}\right)\left(y-m_{y}\right)}{\sqrt{\Sigma {\left(x-m_{x}\right)}^{2}{\left(y-m_{y}\right)}^{2}}}$$

where *n* is the total numbers of data. *y_i_* and *y_i_*′ represent the measured V_oc_ and the predicted V_oc_, respectively. *ŷ* and *ŷ*′ are the averages of the measured V_oc_ and the predicted V_oc_, respectively. *m_x_* and *m_y_* stand for the means of the *x* variable and the *y* variable, respectively. The higher *r* value indicates that the predicted V_oc_ is in good agreement with the measured V_oc_. When the predicted V_oc_ is in perfect agreement with the measured V_oc_, the *RMSE* is close to 0, and the *r* is close to 1, which reveals the model is better.

### 2.4. Visual Interpretation

SHAP originates from cooperative game theory and is gradually applied to explain various ML models, which makes the ML model not to be a “black-box model”. SHAP is an accumulated interpretive tool. The biggest advantage of SHAP is that it reflects the impact of each input feature for the output feature and also shows the negative or positive contribution of each input feature. In this work, we made three types of explanations for SHAP. (1) Global interpretation: The importance of all input features is sorted, and the contribution of each input feature for the output target (the predicted V_oc_) is visualized. (2) Local explanations: explaining the impact of features on the predicted V_oc_ under a single feature or two feature interactions. (3) Sample interpretation: the explanatory analysis of the main input features for the individual sample predictive value in the output target (the predicted V_oc_).

## 3. Result and Discussions

### 3.1. The Influence FMOs of Donor and Acceptor on V_oc_

**Figure 2:**
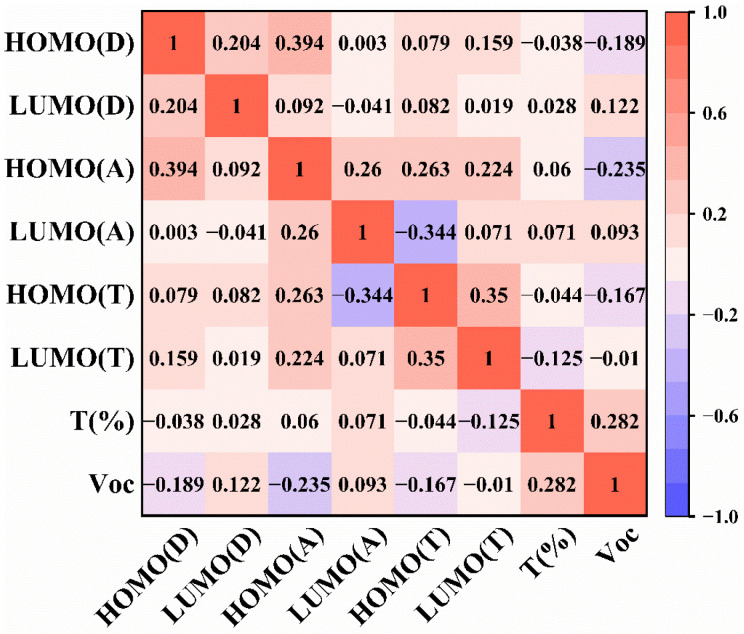
Pearson correlation matrix between V_oc_ and input features. (The value of the correlation coefficient *r* represents the direction and strength of the linear relationship between the input and output feature, which range from −1 to +1, and the corresponding color is from blue to red.)

To analyze the correlation between FMOs of the donor and acceptor and the V_oc_ in ternary PSCs, the Pearson correlation coefficient is calculated to measure the linear relationship between the input feature and output feature. As shown in Figure 2, it is not difficult to find that there is the highest correlation between V_oc_ and the doping concentration T_(%)_ of the third component (*r*_T(%)_ = 0.282). Therefore, it is essential to focus on T_(%)_ of the third component for increasing the V_oc_ of the ternary PSCs. Tao Wang’s team reported that V_oc_ gradually increased with the doping ratio concentration of IDMIC-4F into the PBDB-T-2F:BTP-4F binary PSCs. When the binary system PBDB-T-2F:BTP-4F has 0%, 5%, 10%, 15% and 20% of IDMIC-4F, respectively, the V_oc_ of the device is 0.855 V, 0.858 V, 0.864 V, 0.867 V, 0.876 V and 0.890 V, respectively. The maximum PCE of 16.6% can be achieved with the V_oc_ of 0.864 V, short circuit current (J_sc_) density of 25.8 mA cm^−2^ and the Fill factor (FF) of 74.4% [29]. Increasing a third component can reduce non-radiative voltage loss in ternary PSCs, resulting in the higher V_oc_ in devices [30,31]. More importantly, introducing a third component can significantly optimize the morphology of the active layer in ternary PSCs, which simultaneously promotes J_sc_, V_oc_ and FF [32,33]. Moreover, no matter whether it is the donor or the acceptor, the HOMO energy level shows the negative correlation with V_oc_, while the LUMO energy level is positively correlated with V_oc_ in Figure 2. They reveal that the HOMO energy level of the donor (HOMO_(D)_) should be appropriately reduced or the LUMO energy level of the acceptor (HOMO_(A)_) should be increased to obtain a high V_oc_ of ternary PSCs. On this basis, the introduction of the third component changes the arrangement of the energy level in the donor and acceptor, which can promote the exciton dissociation, improve the charge mobility and inhibit the recombination of the active layer to gain high V_oc_ in ternary PSCs [34,35]. In addition, the correlation between V_oc_ and the HOMO and LUMO of the third component (HOMO_(T)_ and LUMO_(T)_) indicates that reducing HOMO_(T)_ and LUMO_(T)_ is beneficial to increase V_oc_. The reason for this may be that the cascaded energy level alignment of ternary PSCs accelerates the generated exciton dissociation process by multiple interfaces (Donor—Third component, Acceptor—Third component, Donor—Acceptor) to achieve a high V_oc_ [36].

**Figure 3:**
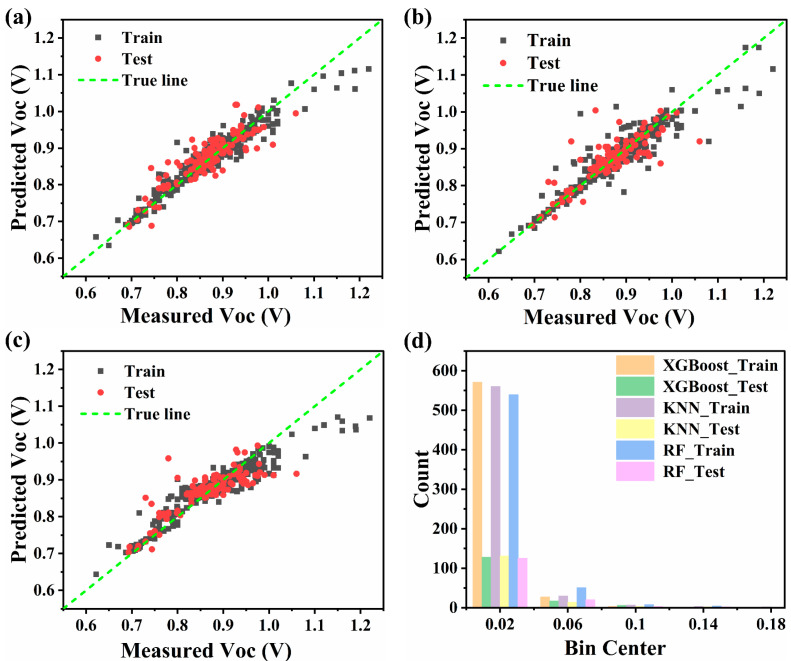
The relationship between the predicted V_oc_ and measured V_oc_ based on different regression algorithms (**a**) XGBoost, (**b**) KNN and (**c**) RF (black dots represent training data; red dots stand for testing data, and when data points fall on green dash lines, it means that the predicted and measured V_oc_ are equal), (**d**) Histogram of frequency distribution of fitting error in the XGBoost, KNN and RF models.

To assess the accuracy of the model, the XGBoost, KNN and RF algorithms based on the FMOs dataset are used to build the V_oc_ prediction model. As seen in Figure 3, the three ML prediction models display that there is no significant outlier in the prediction results, indicating the potential of the models for predicting the V_oc_ of ternary PSCs based on FMOs. Additionally, XGBoost and RF exhibit better accuracy than KNN in Figure 3a–c. In addition, Figure 3d shows the deviation from the predicted V_oc_ to the measured V_oc_ in three ML prediction models. It is clear that the primary counts of the deviation concentrate between 0 and 0.04 V, and deviations above 0.1 V are rare. They reveal that the predicted value of V_oc_ is highly consistent with the measured value. Notably, the XGBoost, KNN and RF prediction models do not show overfitting, and the detailed performance evaluation is shown in Figure S1 of the Supporting Information. The XGBoost model has the best performance on the test set with *RMSE* (0.031), *r* (0.884), and *MAE* (0.022) at their best values, with an exception of *MAPE*(0.025), which is higher in XGBoost than in kNN(0.021), indicating that the XGBoost model has a great advantage in predicting V_oc_. Therefore, the XGBoost model is selected for further research.

**Figure 4:**
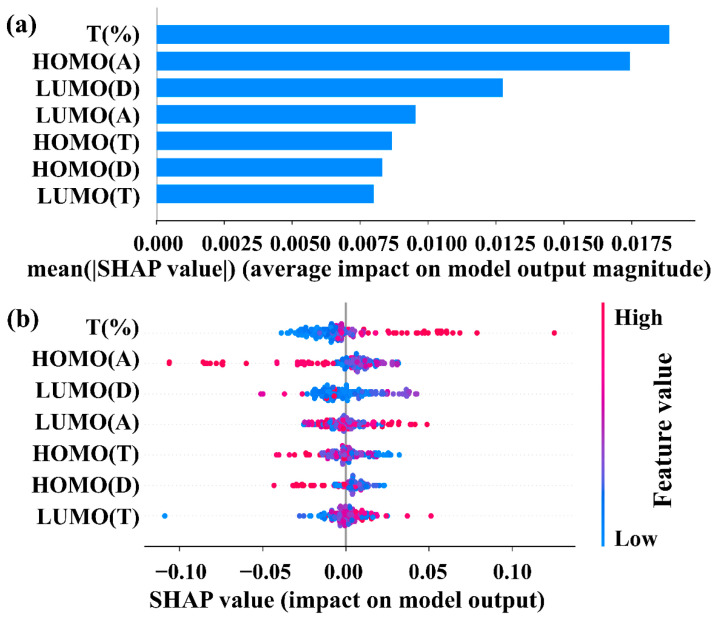
(**a**) Ranking of input feature importance, (**b**) SHAP value summary plot for the FMOs of each material based on XGBoost algorithm.

In order to probe the underlying relationship between V_oc_ and the FMOs of each material in the active layer of ternary PSCs, the SHAP method is employed based on the XGBoost algorithm to analyze the input feature importance and explain the contribution of each input feature for V_oc_. Figure 4a displays the importance score of all input features. It is easy to find that T_(%)_ of the third component is the top factor to affect the V_oc_ of ternary PSCs with non-fullerene, and the second factor is HOMO_(A)_. In Figure 4b, the density scatter plot based on the SHAP interpreter reveals the contribution of each input feature on the V_oc_. Red stands for high eigenvalues, while low eigenvalues are represented by blue color. When the T_(%)_ of the third component increases, the SHAP value increases in a positive direction, while HOMO_(A)_ is the opposite, which is consistent with the previous conclusion in Figure 2. A deeper-lying HOMO level of the main acceptor favors lower energy losses to obtain a higher V_oc_ in ternary PSCs [37,38,39]. Therefore, the main acceptor materials with low HOMO levels should be selected to improve the V_oc_ of the ternary PSCs with two acceptors.

**Figure 5:**
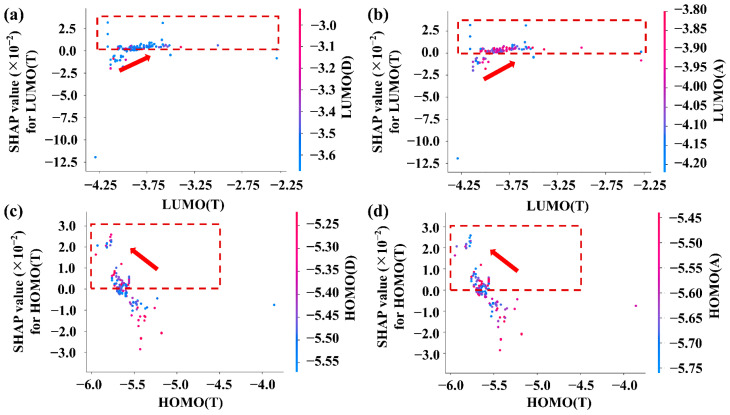
(**a**) The dependent relationship on LUMO_(D)_ and its SHAP values with the changes of LUMO_(T)_, (**b**) the dependent relationship on LUMO_(A)_ and its SHAP values with the changes of LUMO_(T)_, (**c**) the dependent relationship on HOMO_(D)_ and its SHAP values with the changes of HOMO_(T)_ and (**d**) the dependent relationship on HOMO_(A)_ and its SHAP values with the changes of HOMO_(T)_.

To investigate the impact of a single input feature on the V_oc_ of ternary PSCs, the SHAP dependency relationship is utilized to analyze the contribution of a single input feature to the prediction results. It should be noted that SHAP emphasizes single sample interpretation, and the contribution of each input feature is not directly related to the amount of data. Figure 4 illustrates the results of the analysis on the testing set. The red dotted box denotes the area with a positive SHAP value, which stands for promoting V_oc_. Additionally, each point is colored to highlight the interaction with input feature. Figure 5a,b reveal that higher-lying LUMO_(T)_ has more of a contribution to V_oc_, while Figure 5c,d show that the V_oc_ has an upward trend with the lower-lying HOMO_(T)_. However, all changed values of LUMO and HOMO in the third component must be appropriate. As shown in Figure 5a,b, most of the SHAP values are in the positive range, resulting in the increased V_oc_ with the high-level LUMO_(T)_, but there are not too many changes of V_oc_ with the gradually increased LUMO_(T)_. This underscores the significance of energy level matching for achieving an effective improvement in V_oc_ of ternary PSCs [40]. Similarly, as shown in Figure 5c,d, the same trend happens for the effect of the deeper HOMO_(T)_ on V_oc_ of ternary PSCs. More importantly, the color rendering from Figure 5 shows that the co-effect of the HOMO energy level of the third component (HOMO_(T)_) with HOMO_(D)_ and HOMO_(A)_ on V_oc_ being greater than that of LUMO_(T)_ with the LUMO energy level of the donor (LUMO_(D)_) and the LUMO energy level of the acceptor (LUMO_(A)_) on V_oc_. Therefore, V_oc_ in ternary PSCs has a high relationship with the third component, because it changes the matched energy level of the donor and acceptor, leading to the reduction of the offset between the energy levels of donor and acceptor. Lower-lying HOMO_(T)_ is conducive to the construction of the D:A1:T cascaded energy level structure, while higher-lying LUMO_(T)_ reduces the band gap for increasing V_oc_ in ternary PSCs [41]. Additionally, it is interesting that the main positive SHAP value of HOMO_(T)_ is around (−5.7 ± 0.1) eV and the most positive SHAP value of LUMO_(T)_ is around (−3.6 ± 0.1) eV. Kuibao Yu et al. selected Y6-1O as the third component, which has a similar molecular structure and energy level as the Y6 acceptor, and the HOMO/LUMO energy levels of Y6 and Y6-1O are −5.71/−4.10 eV and −5.71/−3.84 eV, respectively. Due to the high compatibility between the two acceptors, the third component Y6-1O is completely embedded in the main acceptor Y6 to form an alloying phase. It makes the energy transfer effect between them significantly enhanced. The V_oc_ of ternary PSCs is increased with the additive of Y6-1O content, which is attributed to the fact that the optimized ternary PSCs enhance charge mobility and inhibit charge recombination [42]. The given energy level range of the third component (HOMO_(T)_) is around (−5.7 ± 0.1) eV. LUMO_(T)_ is about (−3.6 ± 0.1) eV and can provide guidance to screen, design and synthesize suitable non-fullerene materials for ternary PSCs.

**Figure 6:**
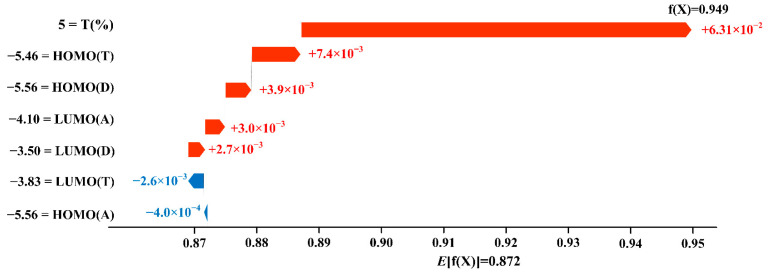
The waterfall diagram of the predicted V_oc_ for PBDB-T-2F:BTP-4F:IDMIC-4F.

To explain the predicted V_oc_ value with the synergistic effect of each input feature, the waterfall diagram in Figure 6 with the example of ternary PSCs based on PBDB-T-2F:BTP-4F:IDMIC-4F is applied to identify the quantitative contribution of each input feature to V_oc_ [29]. The prediction results of the model are driven from the base value to the final model output according to the contribution of each input feature. The input features to increase the predicted value are shown in red, indicating a positive effect, while the input features to reduce the prediction are shown in blue, meaning a negative effect. It is not difficult to find that the input features of HOMO_(A)_ and LUMO_(T)_ reduce the V_oc_ value, and the rest of the input features promote the improvement of V_oc_ with the different degrees. Remarkably, T_(%)_ is the most important factor for improving V_oc_. The SHAP values of the main acceptor’s HOMO and the third component’s LUMO are (−4.0 × 10^−4^) eV and (−2.6 × 10^−3^) eV, respectively. It may be because HOMO_(T)_ (−5.46 eV) is higher than (−5.7 ± 0.1) eV so that it did not increase V_oc_ synergistically with HOMO_(A)_, while LUMO_(T)_ is lower (−3.83 eV), which is consistent with the previous conclusion in Figure 5 [43]. The predicted results also partly explain the synergistically effect of each input feature on the V_oc_ of ternary PSCs.

### 3.2. The Effect of MDs and MFs on V_oc_

**Figure 7:**
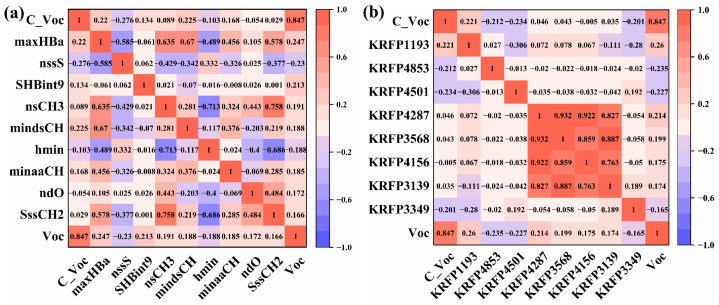
(**a**) Pearson correlation matrix of V_oc_ and E-state MDs and (**b**) Klekota–Roth MFs.

Since the energy level of third component is very important for increasing the V_oc_ in ternary PSCs and the energy level of material is highly dependent on the chemical structure of material, the electronic topological state (E-state) MDs and Klekota–Roth MFs of the third component materials are used to analyze the influence of chemical information of the third component materials on V_oc_. Firstly, the method of feature dimensionality reduction is used to select input features for avoiding the overfitting of ML, and the selected features are displayed in Figure 7a,b. The control the open circuit voltage (C_V_oc_) is the V_oc_ of PSCs without the third component, and it has the highest correlation with V_oc_ (*r*_C_Voc_ = 0.847), which has a positive effect on the V_oc_ improvement of the ternary PSCs. It reveals that introducing a third component to binary devices with high V_oc_ is more likely to obtain a higher V_oc_ in ternary PSCs. The selected 10 features of electrical topological states are described in Table S3. Moreover, the correlations between maxHB, SBint9 and V_oc_ are 0.247 and 0.213, respectively, as depicted in Figure 7a, which suggests that the third component should have strong hydrogen bonding to enhance the V_oc_ of ternary PSCs. Hydrogen bond strength can alter the morphology of the active layer and inhibit the bimolecular recombination for greatly improving V_oc_ [44]. Furthermore, the correlation between nsCH3 and V_oc_ is 0.191, indicating that a certain number of methyl groups plays a critical role in enhancing V_oc_. Notably, hydrogen atoms in the methyl group can form hydrogen bonds. However, their low electronegativity renders the hydrogen bonds strength in the methyl group to be relatively weak compared with the hydrogen bond strength with other atoms [45]. Therefore, comprehensively considering the interaction of hydrogen bond strength and the number of methyl groups of the third component is crucial for enhancing V_oc_ in ternary PSCs.

**Figure 8:**
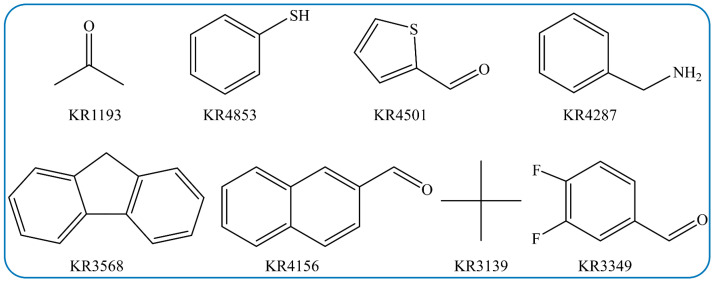
The screened 10 Klekota–Roth MF features.

In addition, eight key input features of the Klekota–Roth MFs with the strong effect on V_oc_ are chosen. As seen in Figure 7b, among the selected fragments, KR1193 containing a carbonyl group is identified as the most influential feature on V_oc_. KR1193, KR4501, KR4156 and KR3349 in Klekota–Roth MFs contain the carbonyl structures, which are consistent with carbonyl characteristics from the E-state MDs. Their descriptions and structures are shown in Figure 8. It further verifies that the design of carbonyls in the third component molecule is very important for V_oc_. KR4501, KR4287, KR3568 and KR415 represent the aromatic ring connected with other different atoms, respectively. These fragments are used to synthesize new molecules, which have stronger intermolecular π-π packing and better backbone coplanarity, resulting in high V_oc_ [46]. Moreover, these aromatic ring structures also promote electron transfer due to their large polarizability and formation of widely distributed molecular orbitals [47]. All in all, the design of NFA in the third component molecules with the aromatic ring fragments may be an effective strategy for improving V_oc_ in ternary PSCs with dual acceptors.

**Figure 9:**
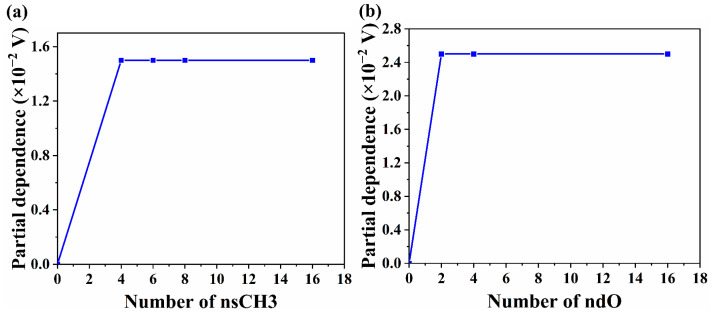
Partial dependency graph of the number of (**a**) methyl groups and (**b**) carbonyl groups.

To evaluate model performance and quantify the effect of functional groups on V_oc_ in the third component to determine the appropriate number of important functional groups in the molecule, we used the XGBoost algorithm to predict the V_oc_ of the ternary PSCs, as shown in Figure S2. The performance evaluation of the XGBoost algorithm is shown in Table S4 ( *RMSE* = 0.031, *MAE* = 0.019, *MAPE* = 0.023 and *r* = 0.832 in the test set of E-state MDs, and *RMSE* = 0.032, *MAE* = 0.019, *MAPE* = 0.022 and *r* = 0.822 in the test set of Klekota–Roth MFs). All results illustrate the feasibility of XGBoost in predicting V_oc_. Moreover, the partial dependence diagrams is utilized to investigate the relationship between the key molecular fragments in the third component and V_oc_, as seen in Figure 9a,b. The results indicate that increasing the number of methyl and carbonyl can enhance the V_oc_ of ternary PSCs. Specifically, with the increase of the number of methyl, the quantitative value of contribution in V_oc_ increases. When the number of methyl is greater than four, the V_oc_ is no longer changed. Jianhui Hou’s team designed BTP-M by introducing a weakly electron-donating methyl group to replace the electron-withdrawing F atom at the terminal group into Y6. BTP-M exhibited a higher LUMO than Y6 and reduced the energy loss in ternary PSCs with PM6:Y6:BTP-M, leading to a higher V_oc_ and larger J_sc_ in devices [36]. Moreover, methyl groups in different positions on the side chains can also cause differences in molecular properties [48]. The number and position of methyl groups are reasonably adjusted to synthesizing the NFA of the third component molecules with high performance and optimizing charge transport in the active layer. More importantly when the number of carbonyl groups is bigger than 2, V_oc_ stays the same. Additionally, carbonyl groups can form non-covalent bonds with other groups, contributing to a better balance between V_oc_ and J_sc_ in ternary PSCs. For example, Xinrui Li et al. found that in the ternary PSCs with PTB7-Th:SR197:ITIC, the carbonyl group of ITIC reacts with the N-H group of SR197 to form N−H…O non-covalent interactions, resulting in an increase in J_sc_ and FF while V_oc_ is almost unchanged, with PCE ranging from 7.92% to 10.29% [49]. Thus, when designing the NFA of the third component molecules to improve the V_oc_ of ternary PSCs, the number of carbonyl groups should be taken into consideration. Moreover, the experimental devices’ V_oc_ values from the publication that is not in datasets are used to verify the predicted V_oc_ by ML in Table S2 in the Supporting Information. It is not difficult to find that the predicted V_oc_ by ML just has a small relative error compared with the measured V_oc_ in the literature which is not in datasets. It reveals that the prediction ML model has certain reliability.

## 4. Conclusions

In this work, we have comprehensively investigated the influence of materials on the V_oc_ of ternary PSCs with dual acceptors by two ways: one is the FMOs of the donor and acceptor, and the other is MDs and MFs in the third component molecules of NFAs. Firstly, three different ML algorithms, XGBoost, KNN and RF, are employed to predict the V_oc_ of ternary PSCs with the energy level of active layer material as input features. In three models, XGBoost has the highest accuracy for predicting the V_oc_ of ternary PSCs, with *RMSE* being 0.031, *MAE* being 0.022, *MAPE* being 0.025 and *r* being 0.884 in the test set. Secondly, the T_(%)_ value of the third component has the most significant impact on V_oc_. It is worth mentioning that the HOMO and LUMO energy levels of the third component should be at (−5.7 ± 0.1) eV and (−3.6 ± 0.1) eV for gaining the maximum V_oc_ value in ternary PSCs with two acceptors. Finally, based on the partial dependence analysis, the hydrogen bond strength and aromatic ring in the third component can enhance V_oc_, and the optimal third component of NFA acceptor molecule should have four methyl groups and two carbonyl groups which should be identified. This work provides design guidance for developing NFA materials and optimizing the V_oc_ value of ternary PSCs.

## Data Availability

The data are available in the Supporting Information.

## Supplementary Materials

The following supporting information can be downloaded at: https://www.mdpi.com/article/10.3390/polym15132954/s1, Figure S1: The evaluation performance (a) *RMSE*, (b) *MAE*, (c) *MAPE* and (d) *r* of the training and testing sets based on three ML algorithms and FMOs as input feature descriptors in Data1. Three ML algorithms are XGBoost, KNN and RF, respectively; Figure S2: The relationship between the predicted V_oc_ and measured V_oc_ values based on the XGBoost model and (a) the E-state MDs and (b) Klekota–Roth MFs as input feature descriptors, respectively; Table S1: The nomenclature and full name of abbreviations, symbols, compounds, etc; Table S2: Device characteristics of the ternary PSCs in literature. And the relative error (%) between the predicted values of V_oc_ based on XGBoost model and measured V_oc_ in literature, which is calculated as = [(V_oc predicted_ - V_oc measured_)/ V_oc measured_]*%; Table S3: The specific meaning of 9 selected molecular 2D E-states; Table S4: The evaluation performance by XGBoost based on 2D E-state MDs and Klekota-Roth MFs; Table S5: Data set of Machine Learning. References [50,51,52,53,54] are cited in the supplementary materials.
