# Supplementary material for: Probing the Effect of Photovoltaic Material on Voc in Ternary Polymer Solar Cells with Non-Fullerene Acceptors by Machine Learning
Source: Polymers (Basel). 2023 Jul 5;15(13):2954. doi: 10.3390/polym15132954 (PMC10346526; doi:10.3390/polym15132954)
Supplement: Supplementary file 1 [file polymers-15-02954-s001.zip › polymers-2441491-supplementary.pdf]

Supporting Information

Probing the Effect of Photovoltaic Material on V_{oc} in Ternary Polymer Solar Cells with Non-Fullerene Acceptors by Machine Learning

Di Huang [1], Zhennan Li [1], Kuo Wang [1], Haixin Zhou [1], Xiaojie Zhao [1], Xinyu Peng [2], Rui Zhang [1], Jipeng Wu [1], Jiaojiao Liang [1,3,*] and Ling Zhao [4,*]

1 College of Railway Transportation, Hunan University of Technology, Zhuzhou 412008, China; dihuang@hut.edu.cn (D.H.); m21085400035@stu.hut.edu.cn (Z.L.); king_20211225@163.com (K.W.); m22085400062@stu.hut.edu.cn (H.Z.); zxj15074980122@163.com (X.Z.); zhang_030720@163.com (R.Z.); jipengwu243@163.com (J.W.)

2 College of Electrical and Information Engineering, Hunan University of Technology, Zhuzhou 412008, China; m22085800061@hut.edu.cn

3 Qinghai Provincial Key Laboratory of Nanomaterials and Nanotechnology, Qinghai Minzu University, Qinghai 810007, China

4 Shandong Provinical Key Laboratory of Optical Communication Science and Technology, School of Physical Science and Information Technology, Liaocheng University, Liaocheng 252059, China

* Correspondence: liangjiaojiao@hut.edu.cn (J.L.); zhaoling9966@163.com (L.Z.)

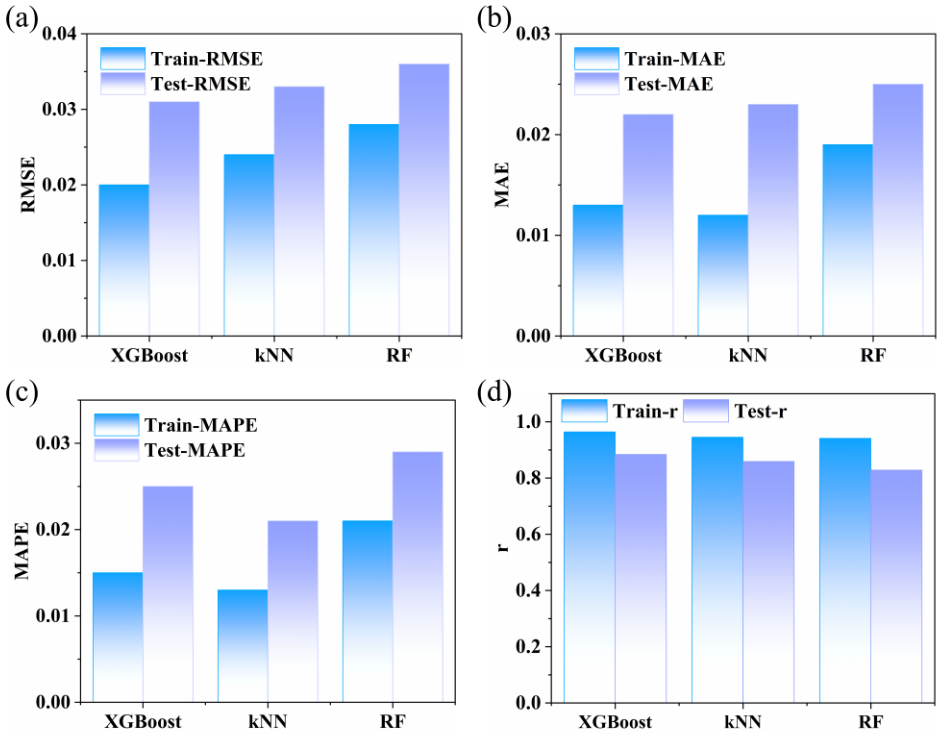

Figure S1. The evaluation performance (a) RMSE, (b) MAE, (c) MAPE and (d) r of the training and testing sets based on three ML algorithms and FMOs as input feature descriptors in Data1. Three ML algorithms are XGBoost, KNN and RF, respectively.

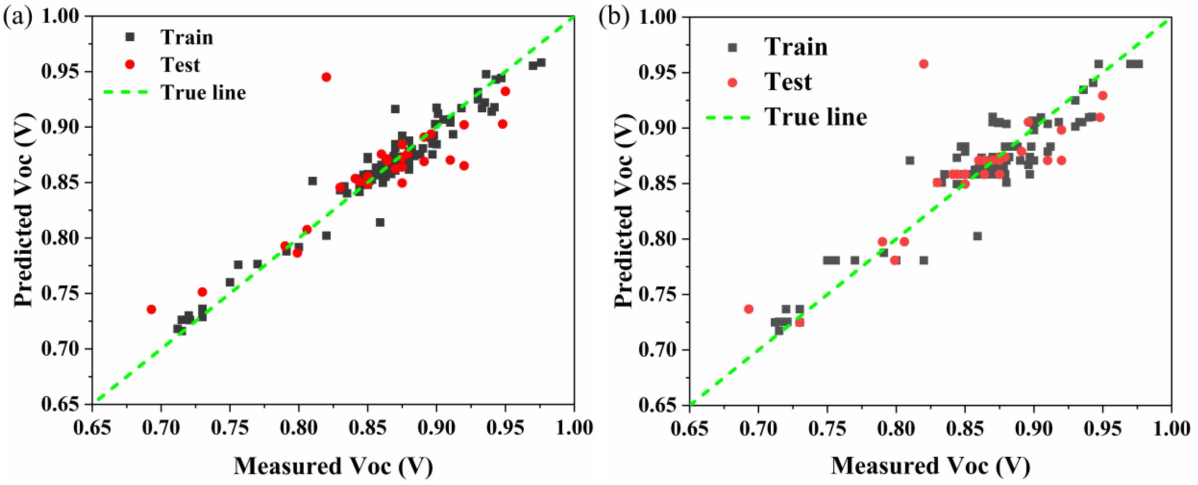

Figure S2. The relationship between the predicted V_{oc} and measured V_{oc} values based on XGBoost model. And (a) the E-states MDs and (b) Klekota-Roth MFs as input feature descriptors, respectively.

Table S1. The nomenclature and full name of abbreviations, symbols, compounds, etc.

Abbreviations, symbols, compounds, etc.	Nomenclature and full name	Abbreviations, symbols, compounds, etc.	Nomenclature and full name
Polymer solar cells	PSCs	Shapley Additive exPlanations	SHAP
Power conversion efficiency	PCE	Partial dependence plots	PDP
Non-fullerene acceptors	NFAs	Pearson correlation coefficient	r
Open circuit voltage	V_{oc}	Root mean square error	RMSE
Short circuit current	J_{sc}	Mean absolute error	MAE
Fill factor	FF	Mean absolute percentage error	MAPE
Density functional theory	DFT	Electronic topological state	E-state
Machine learning	ML	Control the open circuit voltage	C_V_{oc}
K-nearest neighbors	KNN	Molecular descriptors	MDs
Random forest	RF	Molecular fingerprints	MFs
eXtreme gradient boosting	XGBoost	Frontier molecular orbitals	FMOs
the HOMO energy level of the donor	$HOMO_{(D)}$	the LUMO energy level of the donor	$LUMO_{(D)}$
the HOMO energy level of the acceptor	$HOMO_{(A)}$	the LUMO energy level of the acceptor	$LUMO_{(A)}$
the HOMO energy level of the third component	$HOMO_{(T)}$	the LUMO energy level of the third component	$LUMO_{(T)}$

Table S2. Device characteristics of the ternary PSCs in literature. And the relative error (%) between the predicted values of V_{oc} based on XGBoost model and measured V_{oc} in literature, which is calculated as = $[(V_{oc\ predicted} - V_{oc\ measured})/ V_{oc\ measured}]$*%.

Ref	Types of input feature	Active layer	T%	J_{sc} (mA/cm^2	FF (%)	PCE (%)	V_{oc} measured	$V_{oc\ predicted}$ (V)	Relative error (%)

1	FMOs							0.882	2.32
	MDs	PBDB-T:N2200:IDIC	20	12.23	66.2	6.98	0.862	0.876	1.62
	MFs							0.871	1.04
2	FMOs							0.894	1.71
	MDs	PM6:IT-4F:IT-MCA	16.67	20.89	76.0	14.00	0.879	0.869	1.14
	MFs							0.866	1.48
3	FMOs							0.861	0.23
	MDs	PM6:BTP-BO-4F:BTA3	10	26.46	77.87	17.78	0.863	0.870	0.81
	MFs							0.866	0.35
4	FMOs							0.863	0.80
	MDs	PM6:BTP-eC9:ITIC-Th	15	28.00	77.0	18.70	0.870	0.851	2.18
	MFs							0.871	0.11
5	FMOs							0.837	3.01
	MDs	PM6:BTP-eC9:BTP-S16	40	27.73	80.64	19.31	0.863	0.857	0.70
	MFs							0.866	0.35
	FMOs							0.841	3.67
	MDs	PM6:BTP-eC9:BTP-S17	20	27.59	79.55	19.19	0.873	0.858	1.72
	MFs							0.866	0.80

Table S3. The specific meaning of 9 selected molecular 2D E-states.

Features	Description
maxHBa	Maximum E-State for (strong) Hydrogen Bond acceptors
nssS	Count of atom-type E-State: -S-
SHBint9	Sum of E-State descriptors of strength for potential hydrogen
nsCH3	Count of atom-type E-State: $-CH_3$
mindsCH	Minimum atom-type E-State: $-CH_3$
hmin	Minimum H E-State
minaaCH	Minimum atom-type E-State: :CH:
ndO	Count of atom-type E-State: =O
SssCH2	Sum of atom-type E-State: -CH2-

Table S4. The evaluation performance by XGBoost based on 2D E-state MDs and Klekota-Roth MFs.

	Train				Test			
	RMSE	MAE	MAPE	r	RMSE	MAE	MAPE	r
E-states MDs	0.012	0.008	0.010	0.974	0.031	0.019	0.023	0.832
Klekota-Roth MFs	0.018	0.013	0.015	0.936	0.032	0.019	0.022	0.822

Table S5. Data set of Machine Learning.

Data1: HOMO(D), LUMO(D), HOMO(A), LUMO(A), HOMO(T), LUMO(T), T(%) and Device performance parameters (V_{oc}, J_{sc}, FF, PCE) in the data set.														
Donor	Acceptor	Third	$HOMO_{(D)}$	$LUMO_{(D)}$	$HOMO_{(A)}$	$LUMO_{(A)}$	$HOMO_{(T)}$	$LUMO_{(T)}$	$T_{(\%)}$	V_{oc}	J_{sc}	FF	PCE	DOI
PBDB-T-2F	BTP-4F	IDMIC-4F	-5.56	-3.5	-5.65	-4.1	-5.46	-3.83	0	0.855	25.1	73	15.7	10.1007/s11426-019-9681-8
PBDB-T-2F	BTP-4F	IDMIC-4F	-5.56	-3.5	-5.65	-4.1	-5.46	-3.83	5	0.858	25.4	73.1	16.1	10.1007/s11426-019-9681-8
PBDB-T-2F	BTP-4F	IDMIC-4F	-5.56	-3.5	-5.65	-4.1	-5.46	-3.83	10	0.864	25.6	74.2	16.6	10.1007/s11426-019-9681-8
PBDB-T-2F	BTP-4F	IDMIC-4F	-5.56	-3.5	-5.65	-4.1	-5.46	-3.83	15	0.867	24.8	73.9	16.2	10.1007/s11426-019-9681-8
PBDB-T-2F	BTP-4F	IDMIC-4F	-5.56	-3.5	-5.65	-4.1	-5.46	-3.83	20	0.876	24.1	71.8	15.4	10.1007/s11426-019-9681-8
PBDB-T-2F	BTP-4F	IDMIC-4F	-5.56	-3.5	-5.65	-4.1	-5.46	-3.83	100	0.89	16.6	60.5	9.4	10.1007/s11426-019-9681-8
PM6	BTP-4F-C9-20	Y7-BO	-5.5	-3.6	-5.7	-3.9	-5.7	-4.1	0	0.86	26	74.2	16.5	10.1016/j.polymer.2021.124322
PM6	BTP-4F-C9-20	Y7-BO	-5.5	-3.6	-5.7	-3.9	-5.7	-4.1	4.2	0.86	26.3	74.5	16.8	10.1016/j.polymer.2021.124322
PM6	BTP-4F-C9-20	Y7-BO	-5.5	-3.6	-5.7	-3.9	-5.7	-4.1	8.3	0.86	26.8	74.8	17.3	10.1016/j.polymer.2021.124322
PM6	BTP-4F-C9-20	Y7-BO	-5.5	-3.6	-5.7	-3.9	-5.7	-4.1	16.7	0.85	27.2	73.8	17.1	10.1016/j.polymer.2021.124322
PM6	BTP-4F-C9-20	Y7-BO	-5.5	-3.6	-5.7	-3.9	-5.7	-4.1	100	0.84	26.7	73.1	16.4	10.1016/j.polymer.2021.124322
PBDB-T-2F	IT-4F	Y6	-5.5	-3.56	-5.66	-4.14	-5.7	-4.1	0	0.82	20.49	65.22	10.59	10.7498/aps.69.20200624
PBDB-T-2F	IT-4F	Y6	-5.5	-3.56	-5.66	-4.14	-5.7	-4.1	10	0.82	20.76	65.21	11.05	10.7498/aps.69.20200624
PBDB-T-2F	IT-4F	Y6	-5.5	-3.56	-5.66	-4.14	-5.7	-4.1	20	0.83	22.09	68.45	12.48	10.7498/aps.69.20200624
PBDB-T-2F	IT-4F	Y6	-5.5	-3.56	-5.66	-4.14	-5.7	-4.1	30	0.82	21.63	66.43	11.82	10.7498/aps.69.20200624
PM6	Y6	IDTP-4F	-5.48	-3.59	-5.66	-4.1	-5.53	-3.99	0	0.842	25.7	73.5	15.9	10.1016/j.orgel.2021.106201
PM6	Y6	IDTP-4F	-5.48	-3.59	-5.66	-4.1	-5.53	-3.99	8.3	0.863	25.7	77.2	17.1	10.1016/j.orgel.2021.106201
PM6	Y6	IDTP-4F	-5.48	-3.59	-5.66	-4.1	-5.53	-3.99	25	0.876	25	75.8	16.6	10.1016/j.orgel.2021.106201
PM6	Y6	IDTP-4F	-5.48	-3.59	-5.66	-4.1	-5.53	-3.99	41.7	0.883	23.8	71.1	15	10.1016/j.orgel.2021.106201
PM6	Y6	IDTP-4F	-5.48	-3.59	-5.66	-4.1	-5.53	-3.99	58.3	0.893	23.3	70.5	14.7	10.1016/j.orgel.2021.106201
PM6	Y6	IDTP-4F	-5.48	-3.59	-5.66	-4.1	-5.53	-3.99	75	0.916	22.3	68.2	14	10.1016/j.orgel.2021.106201
PM6	Y6	IDTP-4F	-5.48	-3.59	-5.66	-4.1	-5.53	-3.99	100	0.931	20.3	67.3	12.7	10.1016/j.orgel.2021.106201
PTB7-Th	IEICO-4F	T2-ORH	-5.22	-3.64	-5.44	-4.19	-5.66	-3.59	0	0.7	22.07	68	10.53	10.1002/solr.202000742
PTB7-Th	IEICO-4F	T2-ORH	-5.22	-3.64	-5.44	-4.19	-5.66	-3.59	10	0.71	21.75	66	10.4	10.1002/solr.202000742
PTB7-Th	IEICO-4F	T2-ORH	-5.22	-3.64	-5.44	-4.19	-5.66	-3.59	20	0.72	22.28	67	10.77	10.1002/solr.202000742
PTB7-Th	IEICO-4F	T2-ORH	-5.22	-3.64	-5.44	-4.19	-5.66	-3.59	70	0.75	13.14	45	4.44	10.1002/solr.202000742
PTB7-Th	IEICO-4F	T2-ORH	-5.22	-3.64	-5.44	-4.19	-5.66	-3.59	80	0.76	6.46	34	1.66	10.1002/solr.202000742
PTB7-Th	IEICO-4F	T2-ORH	-5.22	-3.64	-5.44	-4.19	-5.66	-3.59	90	0.78	4.33	29	0.98	10.1002/solr.202000742
PTB7-Th	IEICO-4F	T2-ORH	-5.22	-3.64	-5.44	-4.19	-5.66	-3.59	100	1.08	11.28	62	7.51	10.1002/solr.202000742

PTB7-Th	IEICO-4F	T2-OEHRH	-5.22	-3.64	-5.44	-4.19	-5.65	-3.58	5	0.72	21.9	67	10.58	10.1002/solr.202000742
PTB7-Th	IEICO-4F	T2-OEHRH	-5.22	-3.64	-5.44	-4.19	-5.65	-3.58	15	0.73	20.74	70	10.6	10.1002/solr.202000742
PTB7-Th	IEICO-4F	T2-OEHRH	-5.22	-3.64	-5.44	-4.19	-5.65	-3.58	30	0.74	19.76	62	9.14	10.1002/solr.202000742
PTB7-Th	IEICO-4F	T2-OEHRH	-5.22	-3.64	-5.44	-4.19	-5.65	-3.58	40	0.78	16.51	58	7.4	10.1002/solr.202000742
PTB7-Th	IEICO-4F	T2-OEHRH	-5.22	-3.64	-5.44	-4.19	-5.65	-3.58	50	0.79	16.79	52	6.98	10.1002/solr.202000742
PTB7-Th	IEICO-4F	T2-OEHRH	-5.22	-3.64	-5.44	-4.19	-5.65	-3.58	60	0.8	14.06	42	4.68	10.1002/solr.202000742
PTB7-Th	IEICO-4F	T2-OEHRH	-5.22	-3.64	-5.44	-4.19	-5.65	-3.58	100	1.06	11.97	56	7.03	10.1002/solr.202000742
PM6	BTP-Ec9	L8-BO-F	-5.4	-3.48	-5.59	-3.98	-5.59	-3.85	0	0.84	26.62	78.1	17.46	10.1002/adma.202101733
PM6	BTP-Ec9	L8-BO-F	-5.4	-3.48	-5.59	-3.98	-5.59	-3.85	10	0.851	27.11	79.2	18.27	10.1002/adma.202101733
PM6	BTP-Ec9	L8-BO-F	-5.4	-3.48	-5.59	-3.98	-5.59	-3.85	15	0.853	27.35	80	18.66	10.1002/adma.202101733
PM6	BTP-Ec9	L8-BO-F	-5.4	-3.48	-5.59	-3.98	-5.59	-3.85	20	0.854	27.03	79.5	18.35	10.1002/adma.202101733
PM6	BTP-Ec9	L8-BO-F	-5.4	-3.48	-5.59	-3.98	-5.59	-3.85	30	0.855	26.85	77.9	17.88	10.1002/adma.202101733
PM6	BTP-Ec9	L8-BO-F	-5.4	-3.48	-5.59	-3.98	-5.59	-3.85	40	0.862	26.62	77.1	17.69	10.1002/adma.202101733
PM6	BTP-Ec9	L8-BO-F	-5.4	-3.48	-5.59	-3.98	-5.59	-3.85	50	0.874	26.23	75.2	17.24	10.1002/adma.202101733
PM6	BTP-Ec9	L8-BO-F	-5.4	-3.48	-5.59	-3.98	-5.59	-3.85	100	0.936	23.42	76.9	16.82	10.1002/adma.202101733
PBDT-TPD	ITIC-4F	C8IDTT-4Cl	-5.57	-3.59	-5.8	-4.17	-5.76	-4.29	0	0.77	18.17	62.15	8.7	10.1002/cjoc.201900XXX
PBDT-TPD	ITIC-4F	C8IDTT-4Cl	-5.57	-3.59	-5.8	-4.17	-5.76	-4.29	10	0.75	18.76	67.53	9.51	10.1002/cjoc.201900XXX
PBDT-TPD	ITIC-4F	C8IDTT-4Cl	-5.57	-3.59	-5.8	-4.17	-5.76	-4.29	30	0.73	19.44	65.58	9.31	10.1002/cjoc.201900XXX
PBDT-TPD	ITIC-4F	C8IDTT-4Cl	-5.57	-3.59	-5.8	-4.17	-5.76	-4.29	50	0.72	19.85	65.24	9.29	10.1002/cjoc.201900XXX
PBDT-TPD	ITIC-4F	C8IDTT-4Cl	-5.57	-3.59	-5.8	-4.17	-5.76	-4.29	70	0.71	20.18	62.91	9.01	10.1002/cjoc.201900XXX
PBDT-TPD	ITIC-4F	C8IDTT-4Cl	-5.57	-3.59	-5.8	-4.17	-5.76	-4.29	90	0.7	19.14	61	8.16	10.1002/cjoc.201900XXX
PBDT-TPD	ITIC-4F	C8IDTT-4Cl	-5.57	-3.59	-5.8	-4.17	-5.76	-4.29	100	0.67	19.11	58.95	7.55	10.1002/cjoc.201900XXX
J101	MeIC	F-IXIC	-5.3	-3.5	-5.47	-3.82	-5.43	-4	0	0.94	17.8	70.72	11.83	10.1016/j.orgel.2020.105880
J101	MeIC	F-IXIC	-5.3	-3.5	-5.47	-3.82	-5.43	-4	10	0.88	20.04	69.91	12.33	10.1016/j.orgel.2020.105880
J101	MeIC	F-IXIC	-5.3	-3.5	-5.47	-3.82	-5.43	-4	20	0.87	21.89	68.76	13.1	10.1016/j.orgel.2020.105880
J101	MeIC	F-IXIC	-5.3	-3.5	-5.47	-3.82	-5.43	-4	40	0.85	23.6	70.19	14.08	10.1016/j.orgel.2020.105880
J101	MeIC	F-IXIC	-5.3	-3.5	-5.47	-3.82	-5.43	-4	60	0.77	23.66	67.24	12.79	10.1016/j.orgel.2020.105880
J101	MeIC	F-IXIC	-5.3	-3.5	-5.47	-3.82	-5.43	-4	80	0.76	25.62	60.91	12.01	10.1016/j.orgel.2020.105880
J101	MeIC	F-IXIC	-5.3	-3.5	-5.47	-3.82	-5.43	-4	100	0.76	22.09	56.23	9.44	10.1016/j.orgel.2020.105880
J101	MeIC	Cl-IXIC	-5.3	-3.5	-5.47	-3.82	-5.36	-3.98	10	0.884	16.88	62.66	9.35	10.1016/j.orgel.2020.105880
J101	MeIC	Cl-IXIC	-5.3	-3.5	-5.47	-3.82	-5.36	-3.98	20	0.856	18.65	54.84	8.75	10.1016/j.orgel.2020.105880
J101	MeIC	Cl-IXIC	-5.3	-3.5	-5.47	-3.82	-5.36	-3.98	40	0.83	21.95	51.16	9.32	10.1016/j.orgel.2020.105880
J101	MeIC	Cl-IXIC	-5.3	-3.5	-5.47	-3.82	-5.36	-3.98	60	0.8	23.69	46.57	8.83	10.1016/j.orgel.2020.105880

J101	MeIC	Cl-IXIC	-5.3	-3.5	-5.47	-3.82	-5.36	-3.98	80	0.786	22.12	47.95	8.33	10.1016/j.orgel.2020.105880
J101	MeIC	Cl-IXIC	-5.3	-3.5	-5.47	-3.82	-5.36	-3.98	100	0.77	23.12	53.1	9.45	10.1016/j.orgel.2020.105880
PM6	ITIC-4F	BITIC-PhC6F4	-5.5	-3.6	-5.56	-3.61	-5.23	-3.51	0	0.86	20.21	58.31	10.17	10.1002/cssc.202101005
PM6	ITIC-4F	BITIC-PhC6F4	-5.5	-3.6	-5.56	-3.61	-5.23	-3.51	25	0.91	20.09	63.25	11.1	10.1002/cssc.202101005
PM6	ITIC-4F	BITIC-PhC6F4	-5.5	-3.6	-5.56	-3.61	-5.23	-3.51	50	0.96	16.88	42.25	6.75	10.1002/cssc.202101005
PM6	ITIC-4F	BITIC-PhC6F4	-5.5	-3.6	-5.56	-3.61	-5.23	-3.51	100	0.98	13.12	51.92	6.73	10.1002/cssc.202101005
PM6	ITIC-4F	BITIC-C8F4	-5.5	-3.6	-5.56	-3.61	-5.15	-3.33	25	0.9	19.21	64.51	11.1	10.1002/cssc.202101005
PM6	ITIC-4F	BITIC-C8F4	-5.5	-3.6	-5.56	-3.61	-5.15	-3.33	50	0.94	19.5	47.97	8.62	10.1002/cssc.202101005
PM6	ITIC-4F	BITIC-C8F4	-5.5	-3.6	-5.56	-3.61	-5.15	-3.33	75	0.96	16.88	42.25	6.75	10.1002/cssc.202101005
PM6	ITIC-4F	BITIC-C8F4	-5.5	-3.6	-5.56	-3.61	-5.15	-3.33	100	1	14.01	48.59	6.81	10.1002/cssc.202101005
PM6	Y6	BTP-eC9	-5.53	-3.48	-5.69	-3.97	-5.66	-3.93	0	0.84	24.91	71.02	14.87	10.1016/j.dyepig.2022.110083
PM6	Y6	BTP-eC9	-5.53	-3.48	-5.69	-3.97	-5.66	-3.93	20	0.84	24.94	71.55	14.97	10.1016/j.dyepig.2022.110083
PM6	Y6	BTP-eC9	-5.53	-3.48	-5.69	-3.97	-5.66	-3.93	40	0.84	25.68	72.19	15.58	10.1016/j.dyepig.2022.110083
PM6	Y6	BTP-eC9	-5.53	-3.48	-5.69	-3.97	-5.66	-3.93	60	0.85	26.43	72.97	16.39	10.1016/j.dyepig.2022.110083
PM6	Y6	BTP-eC9	-5.53	-3.48	-5.69	-3.97	-5.66	-3.93	80	0.85	25	70.87	15.05	10.1016/j.dyepig.2022.110083
PM6	Y6	BTP-eC9	-5.53	-3.48	-5.69	-3.97	-5.66	-3.93	100	0.86	23.15	69.03	13.27	10.1016/j.dyepig.2022.110083
PTB7-Th	IEICO-4F	P-	-5.24	-3.62	-5.44	-4.19	-5.18	-3.5	0	0.744	19.6	67.08	9.79	10.1016/j.solener.2020.03.008
PTB8-Th	IEICO-4F	P-	-5.24	-3.62	-5.44	-4.19	-5.18	-3.5	15	0.715	21.5	70.78	10.88	10.1016/j.solener.2020.03.008
PTB9-Th	IEICO-4F	P-	-5.24	-3.62	-5.44	-4.19	-5.18	-3.5	25	0.712	19.61	72.22	10.08	10.1016/j.solener.2020.03.008
PTB9-Th	IEICO-4F	P-	-5.24	-3.62	-5.44	-4.19	-5.18	-3.5	100	0.622	3.35	31.45	0.66	10.1016/j.solener.2020.03.008
PM6	Y6	Y-Th2	-5.5	-3.56	-5.7	-4.1	-5.63	-3.63	0	0.841	25.2	70.6	15	10.1016/j.nanoen.2020.104896
PM6	Y6	Y-Th2	-5.5	-3.56	-5.7	-4.1	-5.63	-3.63	5	0.845	25.5	71.1	15.3	10.1016/j.nanoen.2020.104896
PM6	Y6	Y-Th2	-5.5	-3.56	-5.7	-4.1	-5.63	-3.63	10	0.849	25.9	71.4	15.7	10.1016/j.nanoen.2020.104896
PM6	Y6	Y-Th2	-5.5	-3.56	-5.7	-4.1	-5.63	-3.63	15	0.853	26.1	71.8	16	10.1016/j.nanoen.2020.104896
PM6	Y6	Y-Th2	-5.5	-3.56	-5.7	-4.1	-5.63	-3.63	20	0.856	25.2	71.4	15.4	10.1016/j.nanoen.2020.104896
PM6	Y6	Y-BDT	-5.5	-3.56	-5.7	-4.1	-5.51	-3.66	5	0.852	25.4	70.2	15.2	10.1016/j.nanoen.2020.104896
PM6	Y6	Y-BDT	-5.5	-3.56	-5.7	-4.1	-5.51	-3.66	10	0.856	24.9	69.5	14.8	10.1016/j.nanoen.2020.104896
PM6	Y6	Y-BDT	-5.5	-3.56	-5.7	-4.1	-5.51	-3.66	15	0.86	24.5	68.8	14.5	10.1016/j.nanoen.2020.104896
PM6	Y6	Y-BDT	-5.5	-3.56	-5.7	-4.1	-5.51	-3.66	20	0.863	24	68	14.1	10.1016/j.nanoen.2020.104896
PM6	Y6	Y-CDT	-5.5	-3.56	-5.7	-4.1	-5.51	-3.72	5	0.835	25	69	14.4	10.1016/j.nanoen.2020.104896
PM6	Y6	Y-CDT	-5.5	-3.56	-5.7	-4.1	-5.51	-3.72	10	0.832	24.7	67.8	13.9	10.1016/j.nanoen.2020.104896
PM6	Y6	Y-CDT	-5.5	-3.56	-5.7	-4.1	-5.51	-3.72	15	0.827	24.3	67.4	13.6	10.1016/j.nanoen.2020.104896
PM6	Y6	Y-CDT	-5.5	-3.56	-5.7	-4.1	-5.51	-3.72	20	0.822	24.1	66.1	13.1	10.1016/j.nanoen.2020.104896

FTAZ	IDIC	INIC3	-5.38	-3.17	-5.69	-3.91	-5.52	-4.02	0	0.877	15.34	73.3	9.86	10.1016/j.cej.2021.134337
FTAZ	IDIC	INIC3	-5.38	-3.17	-5.69	-3.91	-5.52	-4.02	20	0.878	17	72.3	10.79	10.1016/j.cej.2021.134337
FTAZ	IDIC	INIC3	-5.38	-3.17	-5.69	-3.91	-5.52	-4.02	50	0.873	18.12	72.3	11.44	10.1016/j.cej.2021.134337
FTAZ	IDIC	INIC3	-5.38	-3.17	-5.69	-3.91	-5.52	-4.02	80	0.862	20.22	71.7	12.49	10.1016/j.cej.2021.134337
FTAZ	IDIC	INIC3	-5.38	-3.17	-5.69	-3.91	-5.52	-4.02	100	0.849	20.04	65.9	11.2	10.1016/j.cej.2021.134337
PBDB-T	ITIC	SM-X	-5.2	-3.44	-5.68	-4.06	-5.73	-3.82	0	0.9	16.86	64.71	9.82	10.1002/smll.201905309
PBDB-T	ITIC	SM-X	-5.2	-3.44	-5.68	-4.06	-5.73	-3.82	5	0.909	17.03	64.14	9.94	10.1002/smll.201905309
PBDB-T	ITIC	SM-X	-5.2	-3.44	-5.68	-4.06	-5.73	-3.82	10	0.913	17.18	67.79	10.63	10.1002/smll.201905309
PBDB-T	ITIC	SM-X	-5.2	-3.44	-5.68	-4.06	-5.73	-3.82	15	0.933	16.94	68.72	10.86	10.1002/smll.201905309
PBDB-T	ITIC	SM-X	-5.2	-3.44	-5.68	-4.06	-5.73	-3.82	20	0.935	16.99	68.09	10.82	10.1002/smll.201905309
PBDB-T	ITIC	SM-X	-5.2	-3.44	-5.68	-4.06	-5.73	-3.82	25	0.922	16.7	67.42	10.39	10.1002/smll.201905309
PBDB-T	ITIC	SM-X	-5.2	-3.44	-5.68	-4.06	-5.73	-3.82	30	0.911	15.64	68.77	9.8	10.1002/smll.201905309
PBDB-T	ITIC	SM-Y	-5.2	-3.44	-5.68	-4.06	-5.63	-3.61	5	0.917	16.77	64.82	9.97	10.1002/smll.201905309
PBDB-T	ITIC	SM-Y	-5.2	-3.44	-5.68	-4.06	-5.63	-3.61	10	0.918	16.64	66.09	10.05	10.1002/smll.201905309
PBDB-T	ITIC	SM-Y	-5.2	-3.44	-5.68	-4.06	-5.63	-3.61	15	0.92	16.32	65.51	9.83	10.1002/smll.201905309
PBDB-T	ITIC	SM-Y	-5.2	-3.44	-5.68	-4.06	-5.63	-3.61	20	0.926	16.12	65.3	9.75	10.1002/smll.201905309
PBDB-T	ITIC	SM-Y	-5.2	-3.44	-5.68	-4.06	-5.63	-3.61	25	0.918	16.7	62.86	9.51	10.1002/smll.201905309
PBDB-T	ITIC	SM-Y	-5.2	-3.44	-5.68	-4.06	-5.63	-3.61	30	0.916	15.07	62.22	8.58	10.1002/smll.201905309
PBDB-T	ITIC	SM-XY	-5.2	-3.44	-5.68	-4.06	-5.74	-3.95	5	0.901	16.02	64.88	9.36	10.1002/smll.201905309
PBDB-T	ITIC	SM-XY	-5.2	-3.44	-5.68	-4.06	-5.74	-3.95	10	0.903	15.82	64.58	9.21	10.1002/smll.201905309
PBDB-T	ITIC	SM-XY	-5.2	-3.44	-5.68	-4.06	-5.74	-3.95	15	0.904	15.75	63.38	9.02	10.1002/smll.201905309
PBDB-T	ITIC	SM-XY	-5.2	-3.44	-5.68	-4.06	-5.74	-3.95	20	0.904	15.96	62.07	8.95	10.1002/smll.201905309
PBDB-T	ITIC	SM-XY	-5.2	-3.44	-5.68	-4.06	-5.74	-3.95	25	0.902	15.35	61.29	8.49	10.1002/smll.201905309
PBDB-T	ITIC	SM-XY	-5.2	-3.44	-5.68	-4.06	-5.74	-3.95	30	0.899	15.04	60.22	8.14	10.1002/smll.201905309
PM6	Y6	ITC-2Br1	-5.45	-3.65	-5.65	-4.05	-5.7	-3.95	0	0.83	25.1	74.9	15.6	10.1016/j.orgel.2021.106085
PM6	Y6	ITC-2Br1	-5.45	-3.65	-5.65	-4.05	-5.7	-3.95	8.3	0.84	25.6	76.1	16.4	10.1016/j.orgel.2021.106085
PM6	Y6	ITC-2Br1	-5.45	-3.65	-5.65	-4.05	-5.7	-3.95	12.5	0.85	25.3	77.2	16.6	10.1016/j.orgel.2021.106085
PM6	Y6	ITC-2Br1	-5.45	-3.65	-5.65	-4.05	-5.7	-3.95	16.7	0.86	25	74.2	16	10.1016/j.orgel.2021.106085
PM6	Y6	ITC-2Br1	-5.45	-3.65	-5.65	-4.05	-5.7	-3.95	25	0.87	24.8	70.6	15.2	10.1016/j.orgel.2021.106085
PM6	Y6	ITC-2Br1	-5.45	-3.65	-5.65	-4.05	-5.7	-3.95	41.7	0.9	23.2	67.1	14	10.1016/j.orgel.2021.106085
PM6	Y6	ITC-2Br1	-5.45	-3.65	-5.65	-4.05	-5.7	-3.95	58.3	0.94	21.9	65.6	13.5	10.1016/j.orgel.2021.106085
PM6	Y6	ITC-2Br1	-5.45	-3.65	-5.65	-4.05	-5.7	-3.95	100	1.01	16.6	70	11.7	10.1016/j.orgel.2021.106085
PM6	Y6	C8-DTC	-5.53	-3.54	-5.66	-4.08	-5.6	-3.87	0	0.846	25.6	73.88	16.2	10.1016/j.nanoen.2020.105272

PM6	Y6	C8-DTC	-5.53	-3.54	-5.66	-4.08	-5.6	-3.87	8.3	0.874	26.21	74.58	17.08	10.1016/j.nanoen.2020.105272
PM6	Y6	C8-DTC	-5.53	-3.54	-5.66	-4.08	-5.6	-3.87	10	0.873	26.5	75.61	17.52	10.1016/j.nanoen.2020.105272
PM6	Y6	C8-DTC	-5.53	-3.54	-5.66	-4.08	-5.6	-3.87	12.5	0.873	26.15	75.53	17.24	10.1016/j.nanoen.2020.105272
PM6	Y6	C8-DTC	-5.53	-3.54	-5.66	-4.08	-5.6	-3.87	15	0.871	26.1	75.4	17.14	10.1016/j.nanoen.2020.105272
PM6	Y6	C8-DTC	-5.53	-3.54	-5.66	-4.08	-5.6	-3.87	33.3	0.873	25.37	63.06	13.96	10.1016/j.nanoen.2020.105272
PM6	Y6	C8-DTC	-5.53	-3.54	-5.66	-4.08	-5.6	-3.87	100	0.953	16.87	75.03	12.06	10.1016/j.nanoen.2020.105272
PBDB-T	GL1	F-2Cl	-5.33	-3.53	-5.4	-3.83	-5.76	-3.91	0	0.761	22.59	66.5	11.43	10.1021/acsami.1c13035
PBDB-T	GL1	F-2Cl	-5.33	-3.53	-5.4	-3.83	-5.76	-3.91	20	0.76	23.14	66.5	11.7	10.1021/acsami.1c13035
PBDB-T	GL1	F-2Cl	-5.33	-3.53	-5.4	-3.83	-5.76	-3.91	30	0.756	24.32	67.9	12.5	10.1021/acsami.1c13035
PBDB-T	GL1	F-2Cl	-5.33	-3.53	-5.4	-3.83	-5.76	-3.91	40	0.756	24.54	69.7	12.93	10.1021/acsami.1c13035
PBDB-T	GL1	F-2Cl	-5.33	-3.53	-5.4	-3.83	-5.76	-3.91	50	0.756	24.63	70.7	13.17	10.1021/acsami.1c13035
PBDB-T	GL1	F-2Cl	-5.33	-3.53	-5.4	-3.83	-5.76	-3.91	60	0.753	23.29	71.3	12.52	10.1021/acsami.1c13035
PBDB-T	GL1	F-2Cl	-5.33	-3.53	-5.4	-3.83	-5.76	-3.91	100	0.743	18.92	73.4	10.31	10.1021/acsami.1c13035
PM6	BTP-BO-4Cl	IFL-ED-4F	-5.45	-3.65	-5.66	-4.09	-5.71	-3.84	0	0.84	24.06	71.48	14.4	10.1016/j.jechem.2021.05.032
PM6	BTP-BO-4Cl	IFL-ED-4F	-5.45	-3.65	-5.66	-4.09	-5.71	-3.84	8.3	0.85	25.23	68.54	14.7	10.1016/j.jechem.2021.05.032
PM6	BTP-BO-4Cl	IFL-ED-4F	-5.45	-3.65	-5.66	-4.09	-5.71	-3.84	100	0.94	13.64	58.24	7.5	10.1016/j.jechem.2021.05.032
PM6	BTP-BO-4Cl	IDT-ED-4F	-5.45	-3.65	-5.66	-4.09	-5.38	-3.88	8.3	0.85	24.91	68.01	14.4	10.1016/j.jechem.2021.05.032
PM6	BTP-BO-4Cl	IDT-ED-4F	-5.45	-3.65	-5.66	-4.09	-5.38	-3.88	100	0.88	22.14	53.04	10.4	10.1016/j.jechem.2021.05.032
PM6	BTP-BO-4Cl	IDTT-ED-2F	-5.45	-3.65	-5.66	-4.09	-5.47	-3.81	8.3	0.85	25.16	71.26	15.2	10.1016/j.jechem.2021.05.032
PM6	BTP-BO-4Cl	IDTT-ED-2F	-5.45	-3.65	-5.66	-4.09	-5.47	-3.81	100	0.91	5.1	31.75	1.5	10.1016/j.jechem.2021.05.032
PM6	NCBDT-4Cl	LA1	-5.48	-3.64	-5.63	-4.04	-5.7	-3.91	0	0.827	21.81	74.77	13.48	10.1002/adfm.202007088
PM6	NCBDT-4Cl	LA1	-5.48	-3.64	-5.63	-4.04	-5.7	-3.91	8.3	0.85	22.54	72.89	13.96	10.1002/adfm.202007088
PM6	NCBDT-4Cl	LA1	-5.48	-3.64	-5.63	-4.04	-5.7	-3.91	16.7	0.874	23.58	72.25	14.89	10.1002/adfm.202007088
PM6	NCBDT-4Cl	LA1	-5.48	-3.64	-5.63	-4.04	-5.7	-3.91	23.1	0.897	23.29	73.69	15.39	10.1002/adfm.202007088
PM6	NCBDT-4Cl	LA1	-5.48	-3.64	-5.63	-4.04	-5.7	-3.91	33.3	0.897	22.31	72.69	14.49	10.1002/adfm.202007088
PM6	NCBDT-4Cl	LA1	-5.48	-3.64	-5.63	-4.04	-5.7	-3.91	41.2	0.898	21.71	72.58	14.35	10.1002/adfm.202007088
PM6	NCBDT-4Cl	LA9	-5.48	-3.64	-5.63	-4.04	-5.71	-3.96	8.3	0.838	22.39	74.87	14.04	10.1002/adfm.202007088
PM6	NCBDT-4Cl	LA9	-5.48	-3.64	-5.63	-4.04	-5.71	-3.96	16.7	0.85	23.37	73.7	14.84	10.1002/adfm.202007088
PM6	NCBDT-4Cl	LA9	-5.48	-3.64	-5.63	-4.04	-5.71	-3.96	23.1	0.864	24.42	74.58	15.75	10.1002/adfm.202007088
PM6	NCBDT-4Cl	LA9	-5.48	-3.64	-5.63	-4.04	-5.71	-3.96	33.3	0.865	23.58	73.77	14.99	10.1002/adfm.202007088
PM6	NCBDT-4Cl	LA9	-5.48	-3.64	-5.63	-4.04	-5.71	-3.96	41.2	0.872	22.89	72.73	14.49	10.1002/adfm.202007088
PM6	BTP-eC9	BTP-F	-5.4	-3.48	-5.59	-3.98	-5.55	-3.86	0	0.845	26.81	77.5	17.58	10.1039/d1ee01864g
PM6	BTP-eC9	BTP-F	-5.4	-3.48	-5.59	-3.98	-5.55	-3.86	10	0.854	26.88	78.7	18.07	10.1039/d1ee01864g

PM6	BTP-eC9	BTP-F	-5.4	-3.48	-5.59	-3.98	-5.55	-3.86	15	0.858	26.99	79.7	18.45	10.1039/d1ee01864g
PM6	BTP-eC9	BTP-F	-5.4	-3.48	-5.59	-3.98	-5.55	-3.86	100	0.9	24.05	75	16.2	10.1039/d1ee01864g
PM6	Y6	IDTT-M	-5.28	-3.49	-5.81	-4.5	-5.78	-4.05	0	0.841	25.41	72.45	15.48	10.1002/adfm.202105304
PM6	Y6	IDTT-M	-5.28	-3.49	-5.81	-4.5	-5.78	-4.05	10	0.862	25.6	72.95	16.1	10.1002/adfm.202105304
PM6	Y6	IDTT-M	-5.28	-3.49	-5.81	-4.5	-5.78	-4.05	15	0.872	25.81	73.89	16.63	10.1002/adfm.202105304
PM6	Y6	IDTT-M	-5.28	-3.49	-5.81	-4.5	-5.78	-4.05	20	0.877	24.88	72.64	15.85	10.1002/adfm.202105304
PM6	Y6	IDTT-M	-5.28	-3.49	-5.81	-4.5	-5.78	-4.05	30	0.889	23.68	68	14.32	10.1002/adfm.202105304
PM6	Y6	IDTT-M	-5.28	-3.49	-5.81	-4.5	-5.78	-4.05	100	1.16	11.05	60.7	7.78	10.1002/adfm.202105304
PM6	Y6	PBDTDTzT	-5.5	-3.46	-5.65	-4.1	-5.52	-3.57	0	0.85	25.5	73.5	15.8	10.1016/j.cej.2021.130575
PM6	Y6	PBDTDTzT	-5.5	-3.46	-5.65	-4.1	-5.52	-3.57	8.3	0.86	26.3	74.9	17	10.1016/j.cej.2021.130575
PM6	Y6	PBDTDTzT	-5.5	-3.46	-5.65	-4.1	-5.52	-3.57	15.4	0.87	25.6	70.7	15.7	10.1016/j.cej.2021.130575
PM6	Y6	PBDTDTzT	-5.5	-3.46	-5.65	-4.1	-5.52	-3.57	21.4	0.87	25	71.4	15.5	10.1016/j.cej.2021.130575
PM6	Y6	Y-T	-5.52	-3.66	-5.68	-4.1	-5.42	-3.7	0	0.846	26.37	70.3	15.64	10.1016/j.nanoen.2021.106538
PM6	Y6	Y-T	-5.52	-3.66	-5.68	-4.1	-5.42	-3.7	5	0.85	26.37	72.88	16.28	10.1016/j.nanoen.2021.106538
PM6	Y6	Y-T	-5.52	-3.66	-5.68	-4.1	-5.42	-3.7	10	0.865	26.9	74.97	17.37	10.1016/j.nanoen.2021.106538
PM6	Y6	Y-T	-5.52	-3.66	-5.68	-4.1	-5.42	-3.7	15	0.869	26.28	75.24	17.11	10.1016/j.nanoen.2021.106538
PM6	Y6	Y-T	-5.52	-3.66	-5.68	-4.1	-5.42	-3.7	100	1.19	9.21	46.15	5.06	10.1016/j.nanoen.2021.106538
PTQ10	BTP-Ph	BTP-Th	-5.53	-2.81	-5.58	-3.79	-5.6	-3.78	0	0.888	24.7	77.9	17.1	10.1002/aenm.202100079
PTQ10	BTP-Ph	BTP-Th	-5.53	-2.81	-5.58	-3.79	-5.6	-3.78	8.3	0.888	24.9	78.1	17.3	10.1002/aenm.202100079
PTQ10	BTP-Ph	BTP-Th	-5.53	-2.81	-5.58	-3.79	-5.6	-3.78	16.7	0.888	25.2	78.6	17.6	10.1002/aenm.202100079
PTQ10	BTP-Ph	BTP-Th	-5.53	-2.81	-5.58	-3.79	-5.6	-3.78	33.3	0.886	25.3	77.2	17.3	10.1002/aenm.202100079
PTQ10	BTP-Ph	BTP-Th	-5.53	-2.81	-5.58	-3.79	-5.6	-3.78	100	0.878	25.2	76.2	16.8	10.1002/aenm.202100079
PTQ10	BTP-Ph	BTP-C11	-5.53	-2.81	-5.58	-5.64	-3.86	-3.78	8.3	0.883	24.8	78	17.1	10.1002/aenm.202100079
PTQ10	BTP-Ph	BTP-C11	-5.53	-2.81	-5.58	-5.64	-3.86	-3.78	16.7	0.88	25	78.1	17.2	10.1002/aenm.202100079
PTQ10	BTP-Ph	BTP-C11	-5.53	-2.81	-5.58	-5.64	-3.86	-3.78	33.3	0.876	25	77.6	17	10.1002/aenm.202100079
PM6	Y6	ITIC	-5.54	-3.65	-5.62	-4.11	-5.6	-3.85	0	0.844	24.54	76.06	15.76	10.1021/acs.jpclett.0c03177
PM6	Y6	ITIC	-5.54	-3.65	-5.62	-4.11	-5.6	-3.85	4.2	0.856	25.03	75.67	16.2	10.1021/acs.jpclett.0c03177
PM6	Y6	ITIC	-5.54	-3.65	-5.62	-4.11	-5.6	-3.85	8.3	0.856	25.17	74.58	16.06	10.1021/acs.jpclett.0c03177
PM6	Y6	ITIC	-5.54	-3.65	-5.62	-4.11	-5.6	-3.85	16.7	0.867	24.15	71.12	14.89	10.1021/acs.jpclett.0c03177
PM6	Y6	ITIC	-5.54	-3.65	-5.62	-4.11	-5.6	-3.85	33.3	0.878	23.03	67.86	13.72	10.1021/acs.jpclett.0c03177
PM6	Y6	ITIC	-5.54	-3.65	-5.62	-4.11	-5.6	-3.85	50	0.911	19.74	65.62	11.8	10.1021/acs.jpclett.0c03177
PM6	Y6	ITIC	-5.54	-3.65	-5.62	-4.11	-5.6	-3.85	66.7	0.944	17.74	64.67	10.84	10.1021/acs.jpclett.0c03177
PM6	Y6	ITIC	-5.54	-3.65	-5.62	-4.11	-5.6	-3.85	83.3	0.967	16.25	60.2	9.46	10.1021/acs.jpclett.0c03177

PM6	Y6	ITIC	-5.54	-3.65	-5.62	-4.11	-5.6	-3.85	100	0.978	13.23	55.91	7.23	10.1021/acs.jpclett.0c03177
PBDB-TF	Y6	BDC-4F-C8	-5.65	-3.6	-5.65	-4.1	-5.66	-3.73	0	0.839	25.05	73.03	15.36	10.1002/solr.202100785
PBDB-TF	Y6	BDC-4F-C8	-5.65	-3.6	-5.65	-4.1	-5.66	-3.73	13	0.841	25.05	76.54	16.82	10.1002/solr.202100785
PBDB-TF	Y6	BDC-4F-C8	-5.65	-3.6	-5.65	-4.1	-5.66	-3.73	17	0.847	26.51	74.01	16.3	10.1002/solr.202100785
PBDB-TF	Y6	BDC-4F-C8	-5.65	-3.6	-5.65	-4.1	-5.66	-3.73	33	0.849	25.5	71.32	15.61	10.1002/solr.202100785
PBDB-TF	Y6	BDC-4F-C8	-5.65	-3.6	-5.65	-4.1	-5.66	-3.73	50	0.863	22.2	66.12	12.71	10.1002/solr.202100785
PBDB-TF	Y6	BDC-4F-C8	-5.65	-3.6	-5.65	-4.1	-5.66	-3.73	67	0.868	22.42	62.11	12.12	10.1002/solr.202100785
PBDB-TF	Y6	BDC-4F-C8	-5.65	-3.6	-5.65	-4.1	-5.66	-3.73	83	0.887	20.51	61.2	11.13	10.1002/solr.202100785
PBDB-TF	Y6	BDC-4F-C8	-5.65	-3.6	-5.65	-4.1	-5.66	-3.73	100	0.901	18.97	60.61	10.6	10.1002/solr.202100785
PBDB-T	ITIC	FBR	-5.33	-3.53	-5.54	-3.84	-5.83	-3.75	0	0.88	17.9	62	10.08	10.1002/solr.202000374
PBDB-T	ITIC	FBR	-5.33	-3.53	-5.54	-3.84	-5.83	-3.75	20	0.91	18.55	65	11.17	10.1002/solr.202000374
PBDB-T	ITIC	FBR	-5.33	-3.53	-5.54	-3.84	-5.83	-3.75	40	0.94	17.49	64	10.87	10.1002/solr.202000374
PBDB-T	ITIC	FBR	-5.33	-3.53	-5.54	-3.84	-5.83	-3.75	60	0.96	15.52	61	9.19	10.1002/solr.202000374
PBDB-T	ITIC	FBR	-5.33	-3.53	-5.54	-3.84	-5.83	-3.75	80	1	10.57	56	6.03	10.1002/solr.202000374
PBDB-T	ITIC	FBR	-5.33	-3.53	-5.54	-3.84	-5.83	-3.75	100	1.12	8.62	49	4.8	10.1002/solr.202000374
D18-Cl	N3	Y6	-5.48	-2.75	-5.7	-4.33	-5.65	-4.1	0	0.856	26.9	71.35	16.66	10.1039/d1tc05228d
D18-Cl	N3	Y6	-5.48	-2.75	-5.7	-4.33	-5.65	-4.1	30	0.863	27.07	71.81	17.11	10.1039/d1tc05228d
D18-Cl	N3	Y6	-5.48	-2.75	-5.7	-4.33	-5.65	-4.1	50	0.866	27.89	74.31	17.74	10.1039/d1tc05228d
D18-Cl	N3	Y6	-5.48	-2.75	-5.7	-4.33	-5.65	-4.1	100	0.872	26.38	70.73	16.99	10.1039/d1tc05228d
PTB7-Th	ITIC	TPPI-TPE	-5.24	-3.66	-5.62	-3.92	-5.37	-2.38	0	0.792	16.62	57.88	7.88	10.1016/j.orgel.2020.105827
PTB7-Th	ITIC	TPPI-TPE	-5.24	-3.66	-5.62	-3.92	-5.37	-2.38	5	0.801	17.04	62.62	8.92	10.1016/j.orgel.2020.105827
PTB7-Th	ITIC	TPPI-TPE	-5.24	-3.66	-5.62	-3.92	-5.37	-2.38	10	0.791	17.81	65.63	9.5	10.1016/j.orgel.2020.105827
PTB7-Th	ITIC	TPPI-TPE	-5.24	-3.66	-5.62	-3.92	-5.37	-2.38	15	0.796	17.18	61.68	8.83	10.1016/j.orgel.2020.105827
PTB7-Th	ITIC	TPPI-TPE	-5.24	-3.66	-5.62	-3.92	-5.37	-2.38	20	0.799	16.36	60.45	8.21	10.1016/j.orgel.2020.105827
PTO2	IT-4F	TPPI-TPE	-5.59	-3.67	-5.62	-4.19	-5.37	-2.38	0	0.9	19.78	71.02	12.63	10.1016/j.orgel.2020.105827
PTO2	IT-4F	TPPI-TPE	-5.59	-3.67	-5.62	-4.19	-5.37	-2.38	5	0.9	20.93	67.19	12.78	10.1016/j.orgel.2020.105827
PTO2	IT-4F	TPPI-TPE	-5.59	-3.67	-5.62	-4.19	-5.37	-2.38	10	0.92	22.01	67.22	13.59	10.1016/j.orgel.2020.105827
PTO2	IT-4F	TPPI-TPE	-5.59	-3.67	-5.62	-4.19	-5.37	-2.38	15	0.92	20.33	66.12	12.4	10.1016/j.orgel.2020.105827
PM6	M36	ITCC-M	-5.5	-3.6	-5.62	-3.95	-5.35	-3.69	0	0.87	23	74.8	15	10.1002/adfm.202105794
PM6	M36	ITCC-M	-5.5	-3.6	-5.62	-3.95	-5.35	-3.69	10	0.88	24.5	71.7	15.5	10.1002/adfm.202105794
PM6	M36	ITCC-M	-5.5	-3.6	-5.62	-3.95	-5.35	-3.69	20	0.89	22.97	72.11	14.7	10.1002/adfm.202105794
PM6	BTP-4Cl	PDI-2T	-5.49	-3.68	-5.67	-4.13	-5.75	-4.1	0	0.87	25.48	72.04	15.97	10.1002/adfm.202100316
PM6	BTP-4Cl	PDI-2T	-5.49	-3.68	-5.67	-4.13	-5.75	-4.1	3	0.874	25.7	72.66	16.32	10.1002/adfm.202100316

PM6	BTP-4Cl	PDI-2T	-5.49	-3.68	-5.67	-4.13	-5.75	-4.1	5	0.868	25.43	71.87	15.86	10.1002/adfm.202100316
PM6	BTP-4Cl	PDI-2T	-5.49	-3.68	-5.67	-4.13	-5.75	-4.1	10	0.867	24.92	69.52	15.02	10.1002/adfm.202100316
PM6	DRCN5T	BTP-4Cl	-5.49	-3.68	-5.61	-4.02	-5.67	-4.13	0	0.871	25.48	72.04	15.97	10.1002/adfm.202100316
PM6	DRCN5T	BTP-4Cl	-5.49	-3.68	-5.61	-4.02	-5.67	-4.13	3	0.875	25.85	74.41	16.83	10.1002/adfm.202100316
PM6	DRCN5T	BTP-4Cl	-5.49	-3.68	-5.61	-4.02	-5.67	-4.13	5	0.873	25.5	72.58	16.16	10.1002/adfm.202100316
PM6	DRCN5T	BTP-4Cl	-5.49	-3.68	-5.61	-4.02	-5.67	-4.13	10	0.865	24.86	71.39	15.35	10.1002/adfm.202100316
PM6	Y6	BTIC-EH-	-5.48	-3.65	-5.65	-4	-5.47	-3.69	0	0.84	25.92	75.13	16.35	10.1039/d1ta04454k
PM6	Y6	BTIC-EH-	-5.48	-3.65	-5.65	-4	-5.47	-3.69	5	0.853	26.39	77.9	17.05	10.1039/d1ta04454k
PM6	Y6	BTIC-EH-	-5.48	-3.65	-5.65	-4	-5.47	-3.69	10	0.853	26.39	77.9	17.54	10.1039/d1ta04454k
PM6	Y6	BTIC-EH-	-5.48	-3.65	-5.65	-4	-5.47	-3.69	15	0.872	26.31	75.72	17.37	10.1039/d1ta04454k
PM6	Y6	BTIC-EH-	-5.48	-3.65	-5.65	-4	-5.47	-3.69	20	0.881	25.53	76.33	17.05	10.1039/d1ta04454k
PM6	Y6	BTIC-BO-	-5.48	-3.65	-5.65	-4	-5.48	-3.71	10	0.851	26.02	76.1	16.86	10.1039/d1ta04454k
PM6	Y6	BTIC-Cl	-5.45	-3.65	-5.65	-4.1	-5.69	-3.93	0	0.83	26.03	71.28	15.12	10.1021/acsaem.0c03220
PM6	Y6	BTIC-Cl	-5.45	-3.65	-5.65	-4.1	-5.69	-3.93	7.7	0.85	25.19	70	15.02	10.1021/acsaem.0c03220
PM6	Y6	BTIC-Cl	-5.45	-3.65	-5.65	-4.1	-5.69	-3.93	14.3	0.86	27.1	70.12	16.44	10.1021/acsaem.0c03220
PM6	Y6	BTIC-Cl	-5.45	-3.65	-5.65	-4.1	-5.69	-3.93	20	0.87	25.35	65.72	14.58	10.1021/acsaem.0c03220
PM6	Y6	BTIC-Cl	-5.45	-3.65	-5.65	-4.1	-5.69	-3.93	100	0.99	18.28	69.01	12.47	10.1021/acsaem.0c03220
PBDB-T	IDTO-T-4F	INPIC-Si	-5.28	-3.48	-5.58	-4.04	-5.45	-3.91	0	0.861	20.77	70.18	12.55	10.1016/j.cej.2020.127444
PBDB-T	IDTO-T-4F	INPIC-Si	-5.28	-3.48	-5.58	-4.04	-5.45	-3.91	20	0.866	21.54	72.04	13.44	10.1016/j.cej.2020.127444
PBDB-T	IDTO-T-4F	INPIC-Si	-5.28	-3.48	-5.58	-4.04	-5.45	-3.91	40	0.87	22.55	74.18	14.55	10.1016/j.cej.2020.127444
PBDB-T	IDTO-T-4F	INPIC-Si	-5.28	-3.48	-5.58	-4.04	-5.45	-3.91	60	0.874	21.39	69.78	13.05	10.1016/j.cej.2020.127444
PBDB-T	IDTO-T-4F	INPIC-Si	-5.28	-3.48	-5.58	-4.04	-5.45	-3.91	80	0.882	20.03	67.44	11.91	10.1016/j.cej.2020.127444
PBDB-T	IDTO-T-4F	INPIC-Si	-5.28	-3.48	-5.58	-4.04	-5.45	-3.91	100	0.899	19.76	64.28	11.42	10.1016/j.cej.2020.127444
PM6	Y6	IT-4F	-5.56	-3.5	-5.65	-4.1	-5.69	-4.07	0	0.81	21	66.1	11.29	10.1016/j.dyepig.2020.108613
PM6	Y6	IT-4F	-5.56	-3.5	-5.65	-4.1	-5.69	-4.07	31.8	0.83	22	63.6	11.74	10.1016/j.dyepig.2020.108613
PM6	Y6	IT-4F	-5.56	-3.5	-5.65	-4.1	-5.69	-4.07	50	0.84	22.9	66.1	12.94	10.1016/j.dyepig.2020.108613
PM6	Y6	IT-4F	-5.56	-3.5	-5.65	-4.1	-5.69	-4.07	68.2	0.88	22.1	69.8	14.14	10.1016/j.dyepig.2020.108613
PM6	Y6	IT-4F	-5.56	-3.5	-5.65	-4.1	-5.69	-4.07	100	0.9	17.9	68.5	11.47	10.1016/j.dyepig.2020.108613
PM6	Y6	CTTC-4F-C8	-5.54	-3.61	-5.7	-4.1	-5.63	-3.9	0	0.84	26.76	69.14	15.57	10.1021/acsaem.1c00149
PM6	Y6	CTTC-4F-C8	-5.54	-3.61	-5.7	-4.1	-5.63	-3.9	15	0.85	26.51	70.61	15.91	10.1021/acsaem.1c00149
PM6	Y6	CTTC-4F-C8	-5.54	-3.61	-5.7	-4.1	-5.63	-3.9	100	0.98	19.19	70.79	13.36	10.1021/acsaem.1c00149
PM6	Y6	CTTC-4Cl-C9	-5.54	-3.61	-5.7	-4.1	-5.65	-3.95	15	0.86	27.13	70.85	16.53	10.1021/acsaem.1c00149
PM6	Y6	CTTC-4Cl-C9	-5.54	-3.61	-5.7	-4.1	-5.65	-3.95	25	0.87	25.67	70.82	15.74	10.1021/acsaem.1c00149

PM6	Y6	CTTC-4Cl-C9	-5.54	-3.61	-5.7	-4.1	-5.65	-3.95	50	0.87	25.21	60.4	13.23	10.1021/acsaem.1c00149
PM6	Y6	CTTC-4Cl-C9	-5.54	-3.61	-5.7	-4.1	-5.65	-3.95	75	0.94	22.62	61.32	13.05	10.1021/acsaem.1c00149
PM6	Y6	CTTC-4Cl-C9	-5.54	-3.61	-5.7	-4.1	-5.65	-3.95	100	0.95	20.5	67.23	13.17	10.1021/acsaem.1c00149
PM6	Y6	TIT-2FIC	-5.45	-3.64	-5.6	-4.1	-5.46	-3.98	0	0.857	25.58	73.16	16.04	10.1016/j.cej.2021.131674
PM6	Y6	TIT-2FIC	-5.45	-3.64	-5.6	-4.1	-5.46	-3.98	5	0.866	26.24	73.44	16.69	10.1016/j.cej.2021.131674
PM6	Y6	TIT-2FIC	-5.45	-3.64	-5.6	-4.1	-5.46	-3.98	10	0.87	26.49	74.73	17.22	10.1016/j.cej.2021.131674
PM6	Y6	TIT-2FIC	-5.45	-3.64	-5.6	-4.1	-5.46	-3.98	15	0.873	26.35	73.46	16.9	10.1016/j.cej.2021.131674
PM6	Y6	TIT-2FIC	-5.45	-3.64	-5.6	-4.1	-5.46	-3.98	100	0.905	20.87	69.03	13.05	10.1016/j.cej.2021.131674
PM6	IT-4F	TIT-2FIC	-5.45	-3.64	-5.64	-4.07	-5.46	-3.98	0	0.859	20.38	76.63	13.41	10.1016/j.cej.2021.131674
PM6	IT-4F	TIT-2FIC	-5.45	-3.64	-5.64	-4.07	-5.46	-3.98	30	0.868	21.79	74.73	14.14	10.1016/j.cej.2021.131674
PM6	IT-4F	TIT-2FIC	-5.45	-3.64	-5.64	-4.07	-5.46	-3.98	50	0.875	22.37	73.86	14.46	10.1016/j.cej.2021.131674
PM6	IT-4F	TIT-2FIC	-5.45	-3.64	-5.64	-4.07	-5.46	-3.98	80	0.885	21.09	70.44	13.14	10.1016/j.cej.2021.131674
PM6	IT-4F	TIT-2FIC	-5.45	-3.64	-5.64	-4.07	-5.46	-3.98	100	0.886	19.1	70.06	11.84	10.1016/j.cej.2021.131674
T1	DTSiC-4Cl	DTCC-4Cl	-5.48	-3.63	-5.73	-4.15	-5.71	-4.16	0	1	19.19	70.28	13.49	10.1002/solr.202000357
T1	DTSiC-4Cl	DTCC-4Cl	-5.48	-3.63	-5.73	-4.15	-5.71	-4.16	14.3	0.97	19.62	72.81	13.86	10.1002/solr.202000357
T1	DTSiC-4Cl	DTCC-4Cl	-5.48	-3.63	-5.73	-4.15	-5.71	-4.16	28.6	0.97	19.92	76.24	14.73	10.1002/solr.202000357
T1	DTSiC-4Cl	DTCC-4Cl	-5.48	-3.63	-5.73	-4.15	-5.71	-4.16	50	0.97	19.47	77.71	14.68	10.1002/solr.202000357
T1	DTSiC-4Cl	DTCC-4Cl	-5.48	-3.63	-5.73	-4.15	-5.71	-4.16	71.4	0.97	19.62	74.63	14.2	10.1002/solr.202000357
T1	DTSiC-4Cl	DTCC-4Cl	-5.48	-3.63	-5.73	-4.15	-5.71	-4.16	85.7	0.94	19.29	74.49	13.51	10.1002/solr.202000357
T1	DTSiC-4Cl	DTCC-4Cl	-5.48	-3.63	-5.73	-4.15	-5.71	-4.16	100	0.94	19.69	76.08	14.08	10.1002/solr.202000357
PM6	Y6	AQx-3	-5.58	-3.62	-5.7	-3.9	-5.64	-3.86	0	0.856	25.73	76.8	16.94	10.1002/adma.202100830
PM6	Y6	AQx-3	-5.58	-3.62	-5.7	-3.9	-5.64	-3.86	16.7	0.864	26.53	76.93	17.66	10.1002/adma.202100830
PM6	Y6	AQx-3	-5.58	-3.62	-5.7	-3.9	-5.64	-3.86	33.3	0.87	26.82	77.2	18.01	10.1002/adma.202100830
PM6	Y6	AQx-3	-5.58	-3.62	-5.7	-3.9	-5.64	-3.86	50	0.874	25.42	76.63	17.03	10.1002/adma.202100830
PM6	Y6	AQx-3	-5.58	-3.62	-5.7	-3.9	-5.64	-3.86	100	0.891	24.16	77.4	16.67	10.1002/adma.202100830
PM6	Y6	BTP-M	-5.48	-3.64	-5.65	-4	-5.48	-3.81	0	0.844	24.67	74.96	15.61	10.1039/x0xx00000x
PM6	Y6	BTP-M	-5.48	-3.64	-5.65	-4	-5.48	-3.81	15	0.861	26.59	74.42	16.98	10.1039/x0xx00000x
PM6	Y6	BTP-M	-5.48	-3.64	-5.65	-4	-5.48	-3.81	20	0.875	26.56	73.46	17.03	10.1039/x0xx00000x
PM6	Y6	BTP-M	-5.48	-3.64	-5.65	-4	-5.48	-3.81	25	0.879	25.46	73.21	16.34	10.1039/x0xx00000x
PM6	Y6	BTP-M	-5.48	-3.64	-5.65	-4	-5.48	-3.81	100	0.975	8.43	51.8	4.26	10.1039/x0xx00000x
PM6	Y6	Y6-1O	-5.48	-3.5	-5.71	-4.1	-5.71	-3.84	0	0.847	26.19	77.27	17.16	10.1002/sstr.202100099
PM6	Y6	Y6-1O	-5.48	-3.5	-5.71	-4.1	-5.71	-3.84	10	0.856	26.51	76.61	17.4	10.1002/sstr.202100099
PM6	Y6	Y6-1O	-5.48	-3.5	-5.71	-4.1	-5.71	-3.84	20	0.867	26.74	78.24	18.14	10.1002/sstr.202100099

PM6	Y6	Y6-1O	-5.48	-3.5	-5.71	-4.1	-5.71	-3.84	30	0.867	26.62	77.13	17.8	10.1002/sstr.202100099
PM6	Y6	Y6-1O	-5.48	-3.5	-5.71	-4.1	-5.71	-3.84	50	0.879	26.05	76.44	17.51	10.1002/sstr.202100099
PM6	Y6	Y6-1O	-5.48	-3.5	-5.71	-4.1	-5.71	-3.84	100	0.895	24.79	77.53	17.21	10.1002/sstr.202100099
PM7	IT-4F	PTTtID-Cl	-5.34	-3.66	-5.71	-4.17	-5.53	-4.08	0	0.9	15.8	63.7	9.1	10.1002/cssc.201903087
PM7	IT-4F	PTTtID-Cl	-5.34	-3.66	-5.71	-4.17	-5.53	-4.08	15	0.87	19.6	70.2	12	10.1002/cssc.201903087
PM7	IT-4F	PTTtID-Cl	-5.34	-3.66	-5.71	-4.17	-5.53	-4.08	20	0.86	18.7	65	10.5	10.1002/cssc.201903087
PM7	IT-4F	PTTtID-Cl	-5.34	-3.66	-5.71	-4.17	-5.53	-4.08	50	0.84	18.7	63.4	9.9	10.1002/cssc.201903087
PM7	IT-4F	PTTtID-Cl	-5.34	-3.66	-5.71	-4.17	-5.53	-4.08	80	0.8	15.1	65.1	7.9	10.1002/cssc.201903087
PM7	IT-4F	PTTtID-Cl	-5.34	-3.66	-5.71	-4.17	-5.53	-4.08	100	0.77	13.9	61.5	6.6	10.1002/cssc.201903087
PBDB-T-2Cl	BP-4F	MF1	-5.51	-3.71	-5.68	-3.88	-5.76	-3.82	0	0.88	21.73	76.46	14.62	10.1039/x0xx00000x
PBDB-T-2Cl	BP-4F	MF1	-5.51	-3.71	-5.68	-3.88	-5.76	-3.82	10	0.884	21.89	77.43	14.98	10.1039/x0xx00000x
PBDB-T-2Cl	BP-4F	MF1	-5.51	-3.71	-5.68	-3.88	-5.76	-3.82	20	0.891	22.02	78.05	15.31	10.1039/x0xx00000x
PBDB-T-2Cl	BP-4F	MF1	-5.51	-3.71	-5.68	-3.88	-5.76	-3.82	30	0.895	21.59	78.18	15.11	10.1039/x0xx00000x
PBDB-T-2Cl	BP-4F	MF1	-5.51	-3.71	-5.68	-3.88	-5.76	-3.82	50	0.901	20.58	78.49	14.55	10.1039/x0xx00000x
PBDB-T-2Cl	BP-4F	MF1	-5.51	-3.71	-5.68	-3.88	-5.76	-3.82	100	0.923	17.18	78.61	12.47	10.1039/x0xx00000x
PM6	IM-4F	IDIC	-5.58	-3.73	-5.7	-4.11	-5.63	-4.02	0	0.879	21.91	73.1	14.08	10.1016/j.cej.2021.129539
PM6	IM-4F	IDIC	-5.58	-3.73	-5.7	-4.11	-5.63	-4.02	10	0.891	22.61	78.78	15.86	10.1016/j.cej.2021.129539
PM6	IM-4F	IDIC	-5.58	-3.73	-5.7	-4.11	-5.63	-4.02	20	0.905	21.78	75.59	14.9	10.1016/j.cej.2021.129539
PM6	IM-4F	IDIC	-5.58	-3.73	-5.7	-4.11	-5.63	-4.02	50	0.914	19.44	74.23	13.19	10.1016/j.cej.2021.129539
PM6	IM-4F	IDIC	-5.58	-3.73	-5.7	-4.11	-5.63	-4.02	80	0.935	17.81	73.93	12.31	10.1016/j.cej.2021.129539
PM6	IM-4F	IDIC	-5.58	-3.73	-5.7	-4.11	-5.63	-4.02	100	0.952	16.6	71.35	11.28	10.1016/j.cej.2021.129539
PM6	BTP-eC9	ZY-4Cl	-5.4	-3.48	-5.59	-3.98	-5.64	-3.67	0	0.844	26.84	78.2	17.72	10.1039/D1EE03989J
PM6	BTP-eC9	ZY-4Cl	-5.4	-3.48	-5.59	-3.98	-5.64	-3.67	8	0.859	26.91	78.3	18.1	10.1039/D1EE03989J
PM6	BTP-eC9	ZY-4Cl	-5.4	-3.48	-5.59	-3.98	-5.64	-3.67	12	0.863	27.4	79	18.69	10.1039/D1EE03989J
PM6	BTP-eC9	ZY-4Cl	-5.4	-3.48	-5.59	-3.98	-5.64	-3.67	16	0.865	26.84	78	18.11	10.1039/D1EE03989J
PM6	BTP-eC9	ZY-4Cl	-5.4	-3.48	-5.59	-3.98	-5.64	-3.67	25	0.874	24.95	75.3	16.42	10.1039/D1EE03989J
PM6	BTP-eC9	ZY-4Cl	-5.4	-3.48	-5.59	-3.98	-5.64	-3.67	40	0.898	23.89	72.5	15.56	10.1039/D1EE03989J
PM6	BTP-eC9	ZY-4Cl	-5.4	-3.48	-5.59	-3.98	-5.64	-3.67	70	0.908	20.9	64.1	12.16	10.1039/D1EE03989J
PM6	BTP-eC9	ZY-4Cl	-5.4	-3.48	-5.59	-3.98	-5.64	-3.67	100	1.012	5.12	45.4	2.32	10.1039/D1EE03989J
reg-PThE	ITIC	CDT-TFP	-5.51	-3.46	-5.49	-3.85	-6.09	-3.93	0	0.9	17.69	67.91	10.9	10.1002/cssc.202101824
reg-PThE	ITIC	CDT-TFP	-5.51	-3.46	-5.49	-3.85	-6.09	-3.93	15	0.91	17.38	70.2	11.25	10.1002/cssc.202101824
reg-PThE	ITIC	CDT-TFP	-5.51	-3.46	-5.49	-3.85	-6.09	-3.93	30	0.93	17.96	69.77	11.65	10.1002/cssc.202101824
reg-PThE	ITIC	CDT-TFP	-5.51	-3.46	-5.49	-3.85	-6.09	-3.93	45	0.95	16.22	69.5	10.68	10.1002/cssc.202101824

reg-PThE	ITIC	CDT-TFP	-5.51	-3.46	-5.49	-3.85	-6.09	-3.93	60	0.97	15.62	65.69	9.96	10.1002/cssc.202101824
reg-PThE	ITIC	CDT-TFP	-5.51	-3.46	-5.49	-3.85	-6.09	-3.93	75	1.01	14.34	62.1	8.96	10.1002/cssc.202101824
reg-PThE	ITIC	CDT-TFP	-5.51	-3.46	-5.49	-3.85	-6.09	-3.93	100	1.1	12.43	61.4	8.36	10.1002/cssc.202101824
PM6	Y6	ITIC-M	-5.45	-3.65	-5.6	-4.1	-5.51	-3.91	0	0.83	24.8	68.3	14.03	10.3390/polym13152398
PM6	Y6	ITIC-M	-5.45	-3.65	-5.6	-4.1	-5.51	-3.91	10	0.86	22.99	67.34	13.34	10.3390/polym13152398
PM6	Y6	ITIC-M	-5.45	-3.65	-5.6	-4.1	-5.51	-3.91	20	0.88	22.68	65.21	12.96	10.3390/polym13152398
PM6	Y6	ITIC-M	-5.45	-3.65	-5.6	-4.1	-5.51	-3.91	40	0.91	20.78	64.13	12.16	10.3390/polym13152398
PM6	Y6	ITIC-M	-5.45	-3.65	-5.6	-4.1	-5.51	-3.91	100	1.02	13.27	51.26	6.96	10.3390/polym13152398
PM6	NT-4Cl	NCBDT-4Cl	-5.45	-3.65	-5.89	-3.92	-5.6	-4.02	0	0.929	16.62	74.1	11.44	10.1039/d0tc02778b
PM6	NT-4Cl	NCBDT-4Cl	-5.45	-3.65	-5.89	-3.92	-5.6	-4.02	40	0.899	20.99	77.1	14.55	10.1039/d0tc02778b
PM6	NT-4Cl	NCBDT-4Cl	-5.45	-3.65	-5.89	-3.92	-5.6	-4.02	100	0.833	20.07	72	12.04	10.1039/d0tc02778b
PM6	BTP-4F	BTP-2F	-5.54	-3.72	-5.7	-3.93	-5.67	-3.88	0	0.829	26.4	74.2	16.28	10.1002/aenm.202103735
PM6	BTP-4F	BTP-2F	-5.54	-3.72	-5.7	-3.93	-5.67	-3.88	10	0.84	26.25	74.96	16.58	10.1002/aenm.202103735
PM6	BTP-4F	BTP-2F	-5.54	-3.72	-5.7	-3.93	-5.67	-3.88	30	0.846	26.16	76.25	16.94	10.1002/aenm.202103735
PM6	BTP-4F	BTP-2F	-5.54	-3.72	-5.7	-3.93	-5.67	-3.88	50	0.857	25.58	74.4	16.33	10.1002/aenm.202103735
PM6	BTP-4F	BTP-2F	-5.54	-3.72	-5.7	-3.93	-5.67	-3.88	70	0.865	24.65	72.62	15.57	10.1002/aenm.202103735
PM6	BTP-4F	BTP-2F	-5.54	-3.72	-5.7	-3.93	-5.67	-3.88	90	0.877	23.49	72.01	14.96	10.1002/aenm.202103735
PM6	BTP-4F	BTP-2F	-5.54	-3.72	-5.7	-3.93	-5.67	-3.88	100	0.885	22.68	71.71	14.44	10.1002/aenm.202103735
PM6	ABP6T-4F	CH1007	-5.47	-3.62	-5.56	-3.83	-5.63	-3.96	0	0.88	24.67	73.01	15.81	10.1002/adfm.202104369
PM6	ABP6T-4F	CH1007	-5.47	-3.62	-5.56	-3.83	-5.63	-3.96	16.7	0.87	25.55	73.4	16.24	10.1002/adfm.202104369
PM6	ABP6T-4F	CH1007	-5.47	-3.62	-5.56	-3.83	-5.63	-3.96	33.3	0.86	26.2	73.85	16.59	10.1002/adfm.202104369
PM6	ABP6T-4F	CH1007	-5.47	-3.62	-5.56	-3.83	-5.63	-3.96	50	0.85	26.52	75.87	17.1	10.1002/adfm.202104369
PM6	ABP6T-4F	CH1007	-5.47	-3.62	-5.56	-3.83	-5.63	-3.96	58.3	0.85	25.8	74.94	16.51	10.1002/adfm.202104369
PBDB-T	ITOT-4Cl	F-Br	-5.33	-2.92	-5.49	-3.9	-5.73	-3.88	0	0.775	22.89	70.5	12.5	10.1016/j.nanoen.2020.104988
PBDB-T	ITOT-4Cl	F-Br	-5.33	-2.92	-5.49	-3.9	-5.73	-3.88	40	0.799	24.4	73.3	14.29	10.1016/j.nanoen.2020.104988
PBDB-T	ITOT-4Cl	F-Br	-5.33	-2.92	-5.49	-3.9	-5.73	-3.88	100	0.862	18.05	74	11.52	10.1016/j.nanoen.2020.104988
PBDB-T-2Cl	IT-4F	IXIC-4Cl	-5.51	-3.57	-5.69	-4.07	-5.52	-4.13	0	0.884	19.94	76.4	13.47	10.1039/C9TC06362E
PBDB-T-2Cl	IT-4F	IXIC-4Cl	-5.51	-3.57	-5.69	-4.07	-5.52	-4.13	30	0.847	23.66	74.7	14.96	10.1039/C9TC06362E
PBDB-T-2Cl	IT-4F	IXIC-4Cl	-5.51	-3.57	-5.69	-4.07	-5.52	-4.13	50	0.834	26.67	70.7	13.95	10.1039/C9TC06362E
PBDB-T-2Cl	IT-4F	IXIC-4Cl	-5.51	-3.57	-5.69	-4.07	-5.52	-4.13	100	0.787	21.91	69.5	11.99	10.1039/C9TC06362E
PBDB-T	Y14	SM16	-5.34	-3.51	-5.45	-3.8	-5.56	-3.78	0	0.83	27.31	70.22	15.91	10.1002/adfm.202107756
PBDB-T	Y14	SM16	-5.34	-3.51	-5.45	-3.8	-5.56	-3.78	9.1	0.844	27.49	73.6	17.09	10.1002/adfm.202107756
PBDB-T	Y14	SM16	-5.34	-3.51	-5.45	-3.8	-5.56	-3.78	13.1	0.856	27	71.42	16.52	10.1002/adfm.202107756

PBDB-T	Y14	SM16	-5.34	-3.51	-5.45	-3.8	-5.56	-3.78	16.7	0.862	27.06	72.1	16.82	10.1002/adfm.202107756
PBDB-T	Y14	SM16	-5.34	-3.51	-5.45	-3.8	-5.56	-3.78	23.1	0.875	25.82	70.8	16	10.1002/adfm.202107756
PBDB-T	Y14	SM16	-5.34	-3.51	-5.45	-3.8	-5.56	-3.78	100	0.948	19.15	58.54	10.63	10.1002/adfm.202107756
PM6	F8IC	IT-4F	-5.48	-3.6	-5.48	-3.82	-5.68	-3.75	0	0.76	23	69.5	12.2	10.1016/j.jechem.2021.05.053
PM6	F8IC	IT-4F	-5.48	-3.6	-5.48	-3.82	-5.68	-3.75	10	0.77	23.6	69.6	12.5	10.1016/j.jechem.2021.05.053
PM6	F8IC	IT-4F	-5.48	-3.6	-5.48	-3.82	-5.68	-3.75	20	0.77	24.1	69	12.6	10.1016/j.jechem.2021.05.053
PM6	F8IC	IT-4F	-5.48	-3.6	-5.48	-3.82	-5.68	-3.75	30	0.78	24.6	66.2	12.8	10.1016/j.jechem.2021.05.053
PM6	F8IC	IT-4F	-5.48	-3.6	-5.48	-3.82	-5.68	-3.75	40	0.79	25.3	68.1	13.6	10.1016/j.jechem.2021.05.053
PM6	F8IC	IT-4F	-5.48	-3.6	-5.48	-3.82	-5.68	-3.75	50	0.8	24.9	68.3	13.7	10.1016/j.jechem.2021.05.053
PM6	F8IC	IT-4F	-5.48	-3.6	-5.48	-3.82	-5.68	-3.75	55	0.8	24.4	70.7	13.8	10.1016/j.jechem.2021.05.053
PM6	F8IC	IT-4F	-5.48	-3.6	-5.48	-3.82	-5.68	-3.75	60	0.8	24.1	70	13.6	10.1016/j.jechem.2021.05.053
PM6	F8IC	IT-4F	-5.48	-3.6	-5.48	-3.82	-5.68	-3.75	70	0.81	23.9	69.5	13.5	10.1016/j.jechem.2021.05.053
PM6	F8IC	IT-4F	-5.48	-3.6	-5.48	-3.82	-5.68	-3.75	100	0.96	20.4	73.6	13	10.1016/j.jechem.2021.05.053
PM6	F8IC	Y6	-5.48	-3.6	-5.48	-3.82	-5.67	-3.78	10	0.76	23.5	70.6	12.7	10.1016/j.jechem.2021.05.053
PM6	F8IC	Y6	-5.48	-3.6	-5.48	-3.82	-5.67	-3.78	20	0.77	24.4	69.9	13.1	10.1016/j.jechem.2021.05.053
PM6	F8IC	Y6	-5.48	-3.6	-5.48	-3.82	-5.67	-3.78	30	0.77	24.6	70.4	13.4	10.1016/j.jechem.2021.05.053
PM6	F8IC	Y6	-5.48	-3.6	-5.48	-3.82	-5.67	-3.78	40	0.77	25.2	70.6	13.8	10.1016/j.jechem.2021.05.053
PM6	F8IC	Y6	-5.48	-3.6	-5.48	-3.82	-5.67	-3.78	50	0.77	23.9	72	13.3	10.1016/j.jechem.2021.05.053
PM6	F8IC	Y6	-5.48	-3.6	-5.48	-3.82	-5.67	-3.78	55	0.78	23	70.5	12.6	10.1016/j.jechem.2021.05.053
PM6	F8IC	Y6	-5.48	-3.6	-5.48	-3.82	-5.67	-3.78	60	0.78	22.4	67.7	11.8	10.1016/j.jechem.2021.05.053
PM6	F8IC	Y6	-5.48	-3.6	-5.48	-3.82	-5.67	-3.78	70	0.78	21.8	62	10.5	10.1016/j.jechem.2021.05.053
PM6	F8IC	Y6	-5.48	-3.6	-5.48	-3.82	-5.67	-3.78	100	0.8	19.5	63.9	10	10.1016/j.jechem.2021.05.053
PM6	Y6	ZY-4Cl	-5.54	-3.61	-5.62	-4.11	-5.64	-3.67	0	0.86	25.2	67.5	14.8	10.1002/solr.202100450
PM6	Y6	ZY-4Cl	-5.54	-3.61	-5.62	-4.11	-5.64	-3.67	7.7	0.89	25.2	67.7	15.3	10.1002/solr.202100450
PM6	Y6	ZY-4Cl	-5.54	-3.61	-5.62	-4.11	-5.64	-3.67	14.3	0.9	25.6	64.7	15	10.1002/solr.202100450
PM6	Y6	ZY-4Cl	-5.54	-3.61	-5.62	-4.11	-5.64	-3.67	100	1.16	3.6	38.6	1.6	10.1002/solr.202100450
PBDB-T	BCPT-4F	F-Br	-5.26	-3.63	-5.36	-3.91	-5.73	-3.88	0	0.776	22.96	69.8	12.43	10.1039/d0ta00677g
PBDB-T	BCPT-4F	F-Br	-5.26	-3.63	-5.36	-3.91	-5.73	-3.88	50	0.806	24.88	70.9	14.23	10.1039/d0ta00677g
PBDB-T	BCPT-4F	F-Br	-5.26	-3.63	-5.36	-3.91	-5.73	-3.88	100	0.862	18.05	74	11.52	10.1039/d0ta00677g
PM6	Y6	IDIC	-5.54	-3.61	-5.62	-4.11	-5.69	-3.91	0	0.845	25.49	75.1	16.19	10.1039/d1ta05268c
PM6	Y6	IDIC	-5.54	-3.61	-5.62	-4.11	-5.69	-3.91	4.2	0.859	25.67	76.5	16.86	10.1039/d1ta05268c
PM6	Y6	IDIC	-5.54	-3.61	-5.62	-4.11	-5.69	-3.91	16.7	0.868	24.49	73.1	15.53	10.1039/d1ta05268c
PM6	Y6	IDIC	-5.54	-3.61	-5.62	-4.11	-5.69	-3.91	100	0.944	15.64	72.9	10.77	10.1039/d1ta05268c

PDBD-TF	BTP-eC9	BTP-S2	-5.54	-3.57	-5.66	-4.06	-5.7	-4	0	0.848	26.51	79.21	17.79	10.1039/d1ee02977k
PDBD-TF	BTP-eC9	BTP-S2	-5.54	-3.57	-5.66	-4.06	-5.7	-4	25	0.878	26.78	79.44	18.66	10.1039/d1ee02977k
PDBD-TF	BTP-eC9	BTP-S2	-5.54	-3.57	-5.66	-4.06	-5.7	-4	50	0.89	25.61	78.02	17.86	10.1039/d1ee02977k
PDBD-TF	BTP-eC9	BTP-S2	-5.54	-3.57	-5.66	-4.06	-5.7	-4	75	0.916	24.2	76.11	16.91	10.1039/d1ee02977k
PDBD-TF	BTP-eC9	BTP-S2	-5.54	-3.57	-5.66	-4.06	-5.7	-4	100	0.951	21.62	74.13	15.27	10.1039/d1ee02977k
P125	Y6	DBTBT-IC	-5.45	-3.48	-5.64	-4.08	-5.77	-3.88	0	0.86	17.89	0.64	13.09	10.1039/d1se01748a
P125	Y6	DBTBT-IC	-5.45	-3.48	-5.64	-4.08	-5.77	-3.88	16.7	0.92	24.48	0.73	16.44	10.1039/d1se01748a
P125	Y6	DBTBT-IC	-5.45	-3.48	-5.64	-4.08	-5.77	-3.88	100	1.02	17.89	0.67	12.23	10.1039/d1se01748a
J71	BTP-BO-4Cl	ITIC	-5.4	-3.24	-5.66	-4.09	-5.62	-3.92	0	0.881	22.88	57.79	11.63	10.1039/d0se00605j
J71	BTP-BO-4Cl	ITIC	-5.4	-3.24	-5.66	-4.09	-5.62	-3.92	25	0.903	22.18	63.78	12.73	10.1039/d0se00605j
J71	BTP-BO-4Cl	ITIC	-5.4	-3.24	-5.66	-4.09	-5.62	-3.92	50	0.91	21.62	68.78	13.5	10.1039/d0se00605j
J71	BTP-BO-4Cl	ITIC	-5.4	-3.24	-5.66	-4.09	-5.62	-3.92	60	0.912	21.33	70.31	13.65	10.1039/d0se00605j
J71	BTP-BO-4Cl	ITIC	-5.4	-3.24	-5.66	-4.09	-5.62	-3.92	70	0.914	20.95	70.63	13.52	10.1039/d0se00605j
J71	BTP-BO-4Cl	ITIC	-5.4	-3.24	-5.66	-4.09	-5.62	-3.92	80	0.915	19.06	71.55	12.47	10.1039/d0se00605j
J71	BTP-BO-4Cl	ITIC	-5.4	-3.24	-5.66	-4.09	-5.62	-3.92	100	0.941	15.39	72.34	10.46	10.1039/d0se00605j
PTB7-Th	PTB7-Th	TT-S-4F	-5.24	-3.64	-5.44	-4.09	-5.3	-3.75	0	0.702	24.5	66.9	11.5	10.1002/marc.202000393
PTB7-Th	PTB7-Th	TT-S-4F	-5.24	-3.64	-5.44	-4.09	-5.3	-3.75	5	0.707	24.8	68.2	12	10.1002/marc.202000393
PTB7-Th	PTB7-Th	TT-S-4F	-5.24	-3.64	-5.44	-4.09	-5.3	-3.75	10	0.715	25.5	68.9	12.5	10.1002/marc.202000393
PTB7-Th	PTB7-Th	TT-S-4F	-5.24	-3.64	-5.44	-4.09	-5.3	-3.75	15	0.718	24.2	66	11.4	10.1002/marc.202000393
PBDB-T	ITIC	IT-MCA	-5.33	-3.42	-5.59	-3.91	-5.7	-3.55	0	0.838	19.79	0.74	12.3	10.1039/d1mh00868d
PBDB-T	ITIC	IT-MCA	-5.33	-3.42	-5.59	-3.91	-5.7	-3.55	9.1	0.861	20.29	0.76	13.2	10.1039/d1mh00868d
PBDB-T	ITIC	IT-MCA	-5.33	-3.42	-5.59	-3.91	-5.7	-3.55	16.7	0.879	20.98	0.76	14	10.1039/d1mh00868d
PBDB-T	ITIC	IT-MCA	-5.33	-3.42	-5.59	-3.91	-5.7	-3.55	23.1	0.891	18.73	0.71	11.8	10.1039/d1mh00868d
PBDB-T	ITIC	IT-MCA	-5.33	-3.42	-5.59	-3.91	-5.7	-3.55	33.3	0.899	16.89	0.62	9.4	10.1039/d1mh00868d
PBDB-T	ITIC	IT-MCA	-5.33	-3.42	-5.59	-3.91	-5.7	-3.55	50	0.921	14.31	0.45	6	10.1039/d1mh00868d
PBDB-T	ITIC	IT-MCA	-5.33	-3.42	-5.59	-3.91	-5.7	-3.55	100	1.22	0.63	0.29	0.2	10.1039/d1mh00868d
PM6	BP4T-4F	BP3T-4T	-5.45	-3.65	-5.67	-3.89	-5.7	-3.82	0	0.847	26.02	76.58	16.88	10.1002/solr.202100365
PM6	BP4T-4F	BP3T-4T	-5.45	-3.65	-5.67	-3.89	-5.7	-3.82	20	0.851	25.86	76.63	16.86	10.1002/solr.202100365
PM6	BP4T-4F	BP3T-4T	-5.45	-3.65	-5.67	-3.89	-5.7	-3.82	40	0.857	25.61	77.07	16.91	10.1002/solr.202100365
PM6	BP4T-4F	BP3T-4T	-5.45	-3.65	-5.67	-3.89	-5.7	-3.82	60	0.862	25.06	76.11	16.44	10.1002/solr.202100365
PM6	BP4T-4F	BP3T-4T	-5.45	-3.65	-5.67	-3.89	-5.7	-3.82	100	0.869	23.57	75.04	15.37	10.1002/solr.202100365
PBB-F	IT-4F	Y6-DT-4F	-5.57	-3.57	-5.68	-3.75	-5.67	-4.04	0	0.91	20.98	70.14	13.39	10.1021/acsami.1c23332
PBB-F	IT-4F	Y6-DT-4F	-5.57	-3.57	-5.68	-3.75	-5.67	-4.04	4.8	0.903	22.55	70.5	14.32	10.1021/acsami.1c23332

PBB-F	IT-4F	Y6-DT-4F	-5.57	-3.57	-5.68	-3.75	-5.67	-4.04	10	0.896	23.68	71.82	15.24	10.1021/acsami.1c23332
PBB-F	IT-4F	Y6-DT-4F	-5.57	-3.57	-5.68	-3.75	-5.67	-4.04	20	0.887	22.49	68.47	13.66	10.1021/acsami.1c23332
PBB-F	IT-4F	Y6-DT-4F	-5.57	-3.57	-5.68	-3.75	-5.67	-4.04	30	0.883	22.14	66.61	13.02	10.1021/acsami.1c23332
PBB-F	IT-4F	Y6-DT-4F	-5.57	-3.57	-5.68	-3.75	-5.67	-4.04	100	0.862	22.16	54.71	10.45	10.1021/acsami.1c23332
PBDB-TF	Y6	DTNIF	-5.56	-3.5	-5.65	-4.1	-5.82	-3.92	0	0.849	25.26	71.36	15.31	10.1039/d0ee03378b
PBDB-TF	Y6	DTNIF	-5.56	-3.5	-5.65	-4.1	-5.82	-3.92	8.3	0.858	26.4	73.8	16.73	10.1039/d0ee03378b
PBDB-TF	Y6	DTNIF	-5.56	-3.5	-5.65	-4.1	-5.82	-3.92	16.7	0.87	25.18	71.41	15.63	10.1039/d0ee03378b
PBDB-TF	Y6	DTNIF	-5.56	-3.5	-5.65	-4.1	-5.82	-3.92	25	0.879	24.81	70.11	15.29	10.1039/d0ee03378b
PBDB-TF	Y6	DTNIF	-5.56	-3.5	-5.65	-4.1	-5.82	-3.92	41.7	0.889	23.49	63.36	13.24	10.1039/d0ee03378b
PBDB-TF	Y6	DTNIF	-5.56	-3.5	-5.65	-4.1	-5.82	-3.92	58.3	0.898	21.04	51.14	9.66	10.1039/d0ee03378b
PBDB-TF	Y6	DTNIF	-5.56	-3.5	-5.65	-4.1	-5.82	-3.92	75	0.923	18.01	50.57	8.41	10.1039/d0ee03378b
PBDB-TF	Y6	DTNIF	-5.56	-3.5	-5.65	-4.1	-5.82	-3.92	100	1.01	12.76	56.86	7.31	10.1039/d0ee03378b
PM6	N3	MF1	-5.45	-3.65	-5.7	-3.88	-5.65	-4.05	0	0.83	26.08	74.13	16.04	10.1002/smll.202104215
PM6	N3	MF1	-5.45	-3.65	-5.7	-3.88	-5.65	-4.05	10	0.83	25.97	77.12	16.62	10.1002/smll.202104215
PM6	N3	MF1	-5.45	-3.65	-5.7	-3.88	-5.65	-4.05	15	0.85	25.45	77.51	16.76	10.1002/smll.202104215
PM6	N3	MF1	-5.45	-3.65	-5.7	-3.88	-5.65	-4.05	30	0.85	24.09	75.85	15.53	10.1002/smll.202104215
PM6	N3	MF1	-5.45	-3.65	-5.7	-3.88	-5.65	-4.05	70	0.86	22.63	69.93	13.61	10.1002/smll.202104215
PM6	N3	MF1	-5.45	-3.65	-5.7	-3.88	-5.65	-4.05	100	0.91	16.95	78.7	12.14	10.1002/smll.202104215
PBQx-TF	eC9-2Cl	F-BTA3	-5.41	-2.82	-5.57	-3.74	-5.52	-3.39	0	0.868	25.9	75.6	17.7	10.1002/adma.202102420
PBQx-TF	eC9-2Cl	F-BTA3	-5.41	-2.82	-5.57	-3.74	-5.52	-3.39	8.33	0.872	26.2	80.4	18.4	10.1002/adma.202102420
PBQx-TF	eC9-2Cl	F-BTA3	-5.41	-2.82	-5.57	-3.74	-5.52	-3.39	14.28	0.876	26.4	79.1	18.3	10.1002/adma.202102420
PBQx-TF	eC9-2Cl	F-BTA3	-5.41	-2.82	-5.57	-3.74	-5.52	-3.39	33.3	0.883	24.9	78.9	17.3	10.1002/adma.202102420
PBQx-TF	eC9-2Cl	F-BTA3	-5.41	-2.82	-5.57	-3.74	-5.52	-3.39	50	0.879	26.7	80.9	19	10.1002/adma.202102420
PBQx-TF	eC9-2Cl	F-BTA3	-5.41	-2.82	-5.57	-3.74	-5.52	-3.39	100	1.15	13.2	75.6	17.7	10.1002/adma.202102420
PM6	Y6	ITCPTC	-5.5	-3.61	-5.6	-4.1	-5.65	-3.95	0	0.844	25.328	76.9	16.44	10.1021/acsenergylett.0c01364
PM6	Y6	ITCPTC	-5.5	-3.61	-5.6	-4.1	-5.65	-3.95	5	0.861	25.674	78.8	17.42	10.1021/acsenergylett.0c01364
PM6	Y6	ITCPTC	-5.5	-3.61	-5.6	-4.1	-5.65	-3.95	16.7	0.872	24.318	76.5	16.22	10.1021/acsenergylett.0c01364
PM6	Y6	ITCPTC	-5.5	-3.61	-5.6	-4.1	-5.65	-3.95	100	0.964	17.285	73.8	12.31	10.1021/acsenergylett.0c01364
PBDB-TF	F-2Cl	3TT-OCIC	-5.44	-3.52	-5.74	-3.88	-5.54	-3.96	0	0.875	19.97	73.7	12.87	10.1007/s11426-020-9921-4
PBDB-TF	F-2Cl	3TT-OCIC	-5.44	-3.52	-5.74	-3.88	-5.54	-3.96	20	0.848	23.75	72.7	14.64	10.1007/s11426-020-9921-4
PBDB-TF	F-2Cl	3TT-OCIC	-5.44	-3.52	-5.74	-3.88	-5.54	-3.96	30	0.844	24.92	72.4	15.23	10.1007/s11426-020-9921-4
PBDB-TF	F-2Cl	3TT-OCIC	-5.44	-3.52	-5.74	-3.88	-5.54	-3.96	40	0.83	25.73	68.4	14.6	10.1007/s11426-020-9921-4
PBDB-TF	F-2Cl	3TT-OCIC	-5.44	-3.52	-5.74	-3.88	-5.54	-3.96	50	0.821	25.4	67	13.97	10.1007/s11426-020-9921-4

PBDB-TF	F-2Cl	3TT-OCIC	-5.44	-3.52	-5.74	-3.88	-5.54	-3.96	100	0.785	24.4	65	12.43	10.1007/s11426-020-9921-4
PBDB-T-2F	FOIC	IT-2F	-5.42	-3.57	-5.36	-3.92	-5.64	-4.03	0	0.92	19.73	69.2	12.56	10.1016/j.nanoen.2019.02.038
PBDB-T-2F	FOIC	IT-2F	-5.42	-3.57	-5.36	-3.92	-5.64	-4.03	20	0.91	20.7	69.8	13.15	10.1016/j.nanoen.2019.02.038
PBDB-T-2F	FOIC	IT-2F	-5.42	-3.57	-5.36	-3.92	-5.64	-4.03	25.42	0.908	21.45	70.1	13.65	10.1016/j.nanoen.2019.02.038
PBDB-T-2F	FOIC	IT-2F	-5.42	-3.57	-5.36	-3.92	-5.64	-4.03	30	0.905	21.9	70.4	13.95	10.1016/j.nanoen.2019.02.038
PBDB-T-2F	FOIC	IT-2F	-5.42	-3.57	-5.36	-3.92	-5.64	-4.03	34.6	0.901	21.22	70.2	13.42	10.1016/j.nanoen.2019.02.038
PBDB-T-2F	FOIC	IT-2F	-5.42	-3.57	-5.36	-3.92	-5.64	-4.03	40	0.898	20.56	69.5	12.83	10.1016/j.nanoen.2019.02.038
PBDB-T-2F	FOIC	IT-2F	-5.42	-3.57	-5.36	-3.92	-5.64	-4.03	100	0.855	16.75	67.2	9.57	10.1016/j.nanoen.2019.02.038
PBDB-T-2F	IT-2F	FTTCN	-5.48	-3.69	-5.64	-4.03	-5.56	-3.91	0	0.92	17.76	73.48	12.01	10.1016/j.nanoen.2019.02.038
PBDB-T-2F	IT-2F	FTTCN	-5.48	-3.69	-5.64	-4.03	-5.56	-3.91	20	0.94	18.18	76.03	12.99	10.1016/j.nanoen.2019.02.038
PBDB-T-2F	IT-2F	FTTCN	-5.48	-3.69	-5.64	-4.03	-5.56	-3.91	40	0.954	17.42	74.47	12.37	10.1016/j.nanoen.2019.02.038
PBDB-T-2F	IT-2F	FTTCN	-5.48	-3.69	-5.64	-4.03	-5.56	-3.91	60	0.965	16.67	72.3	11.63	10.1016/j.nanoen.2019.02.038
PBDB-T-2F	IT-2F	FTTCN	-5.48	-3.69	-5.64	-4.03	-5.56	-3.91	80	0.983	15.91	70.65	11.05	10.1016/j.nanoen.2019.02.038
PBDB-T-2F	IT-2F	FTTCN	-5.48	-3.69	-5.64	-4.03	-5.56	-3.91	100	1.01	14.98	69.51	10.52	10.1016/j.nanoen.2019.02.038
PM6	BP5T-4F	CH1007	-5.47	-3.62	-5.63	-3.88	-5.63	-3.96	0	0.888	24.6	76.3	16.7	10.1002/aenm.202003177
PM6	BP5T-4F	CH1007	-5.47	-3.62	-5.63	-3.88	-5.63	-3.96	15	0.879	25.5	76.7	17.2	10.1002/aenm.202003177
PM6	BP5T-4F	CH1007	-5.47	-3.62	-5.63	-3.88	-5.63	-3.96	30	0.854	25.8	76.4	16.8	10.1002/aenm.202003177
PM6	BP5T-4F	CH1007	-5.47	-3.62	-5.63	-3.88	-5.63	-3.96	50	0.844	26.1	76.5	16.9	10.1002/aenm.202003177
PM6	BP5T-4F	CH1007	-5.47	-3.62	-5.63	-3.88	-5.63	-3.96	75	0.821	26.4	75.5	16.4	10.1002/aenm.202003177
PM6	BP5T-4F	CH1007	-5.47	-3.62	-5.63	-3.88	-5.63	-3.96	100	0.817	26.8	72.9	16	10.1002/aenm.202003177
PM6	Y6	IHIC	-5.45	-3.69	-5.58	-4.25	-5.47	-4.08	0	0.86	24.69	70.87	15.11	10.1002/solr.202200070
PM6	Y6	IHIC	-5.45	-3.69	-5.58	-4.25	-5.47	-4.08	16.67	0.81	26.72	70.69	15.28	10.1002/solr.202200070
PM6	Y6	IT-M	-5.55	-3.78	-5.61	-4.27	-5.58	-3.98	0	0.86	23.51	71.88	14.38	10.1016/j.cej.2021.132048
PM6	Y6	IT-M	-5.55	-3.78	-5.61	-4.27	-5.58	-3.98	9.09	0.88	26.57	64.47	15.09	10.1016/j.cej.2021.132048
PM6	Y6	IT-M	-5.55	-3.78	-5.61	-4.27	-5.58	-3.98	23.08	0.84	23.69	69.71	13.89	10.1016/j.cej.2021.132048
PM6	BTP-Ec9	MOIT-M	-5.5	-3.59	-5.65	-3.98	-5.59	-3.88	0	0.84	27	76.1	17.4	10.1039/d2ta03941a
PM6	BTP-Ec9	MOIT-M	-5.5	-3.59	-5.65	-3.98	-5.59	-3.88	8.33	0.85	27.2	76.1	17.7	10.1039/d2ta03941a
PM6	BTP-Ec9	MOIT-M	-5.5	-3.59	-5.65	-3.98	-5.59	-3.88	16.67	0.86	27.3	76.4	18	10.1039/d2ta03941a
PM6	BTP-Ec9	MOIT-M	-5.5	-3.59	-5.65	-3.98	-5.59	-3.88	25	0.87	27.4	77.3	18.5	10.1039/d2ta03941a
PM6	BTP-Ec9	MOIT-M	-5.5	-3.59	-5.65	-3.98	-5.59	-3.88	33.33	0.88	26.6	73.7	17.3	10.1039/d2ta03941a
PM6	BTP-Ec9	MOIT-M	-5.5	-3.59	-5.65	-3.98	-5.59	-3.88	100	1.01	15.8	64.6	10.3	10.1039/d2ta03941a
PM6	Y6	IDIC-C4Ph	-5.48	-3.66	-5.79	-4.11	-5.69	-4.09	0	0.834	25.88	72.23	15.61	10.1016/j.cej.2022.136691
PM6	Y6	IDIC-C4Ph	-5.48	-3.66	-5.79	-4.11	-5.69	-4.09	8.33	0.86	25.53	76.49	16.8	10.1016/j.cej.2022.136691

PM6	Y6	IDIC-C4Ph	-5.48	-3.66	-5.79	-4.11	-5.69	-4.09	12.5	0.864	26.38	77.22	17.81	10.1016/j.cej.2022.136691
PM6	Y6	IDIC-C4Ph	-5.48	-3.66	-5.79	-4.11	-5.69	-4.09	16.67	0.866	26.8	74.85	17.37	10.1016/j.cej.2022.136691
PM6	Y6	IDIC-C4Ph	-5.48	-3.66	-5.79	-4.11	-5.69	-4.09	25	0.901	24.66	73.7	16.45	10.1016/j.cej.2022.136691
PM6	Y6	IDIC-C4Ph	-5.48	-3.66	-5.79	-4.11	-5.69	-4.09	41.67	0.909	23.53	74.24	15.9	10.1016/j.cej.2022.136691
PM6	Y6	IDIC-C4Ph	-5.48	-3.66	-5.79	-4.11	-5.69	-4.09	50	0.909	23.73	69.68	14.95	10.1016/j.cej.2022.136691
PM6	Y6	IDIC-C4Ph	-5.48	-3.66	-5.79	-4.11	-5.69	-4.09	100	0.917	18.64	71.15	12.13	10.1016/j.cej.2022.136691
PM6	Y6	J71	-5.47	-3.59	-5.65	-4.1	-5.4	-3.24	0	0.84	25.24	73.9	15.6	10.1016/j.nanoen.2020.104447
PM6	Y6	J71	-5.47	-3.59	-5.65	-4.1	-5.4	-3.24	5	0.84	25.34	75.1	16	10.1016/j.nanoen.2020.104447
PM6	Y6	J71	-5.47	-3.59	-5.65	-4.1	-5.4	-3.24	10	0.85	25.55	76	16.5	10.1016/j.nanoen.2020.104447
PM6	Y6	J71	-5.47	-3.59	-5.65	-4.1	-5.4	-3.24	15	0.85	25.42	75.1	16.2	10.1016/j.nanoen.2020.104447
PM6	Y6	J71	-5.47	-3.59	-5.65	-4.1	-5.4	-3.24	20	0.85	25.28	73.2	15.7	10.1016/j.nanoen.2020.104447
PM6	Y6	J71	-5.47	-3.59	-5.65	-4.1	-5.4	-3.24	40	0.86	23.49	70.3	14.2	10.1016/j.nanoen.2020.104447
PM6	Y6	J71	-5.47	-3.59	-5.65	-4.1	-5.4	-3.24	80	0.87	21.22	60.2	11.1	10.1016/j.nanoen.2020.104447
PM6	Y6	J71	-5.47	-3.59	-5.65	-4.1	-5.4	-3.24	100	0.87	20.54	53.6	9.6	10.1016/j.nanoen.2020.104447
PM6	Y6	O-ITIC	-5.18	-3.38	-5.63	-4.28	-5.71	-4.15	0	0.85	25.1	73.6	15.8	10.1021/acsaem.1c02067
PM6	Y6	O-ITIC	-5.18	-3.38	-5.63	-4.28	-5.71	-4.15	10	0.88	25.1	74.7	16.5	10.1021/acsaem.1c02067
PM6	Y6	O-ITIC	-5.18	-3.38	-5.63	-4.28	-5.71	-4.15	20	0.88	23.4	75.1	15.5	10.1021/acsaem.1c02067
PM6	Y6	O-ITIC	-5.18	-3.38	-5.63	-4.28	-5.71	-4.15	30	0.9	23.3	73	15.3	10.1021/acsaem.1c02067
PM6	Y6	O-ITIC	-5.18	-3.38	-5.63	-4.28	-5.71	-4.15	50	0.92	22.3	72.1	14.8	10.1021/acsaem.1c02067
PM6	Y6	O-ITIC	-5.18	-3.38	-5.63	-4.28	-5.71	-4.15	80	0.93	20.4	66.1	12.6	10.1021/acsaem.1c02067
PM6	Y6	O-ITIC	-5.18	-3.38	-5.63	-4.28	-5.71	-4.15	100	0.97	16.8	68.7	11.1	10.1021/acsaem.1c02067
PM6	Y6	BTP-MCA	-5.46	-3.6	-5.65	-4.08	-5.48	-3.58	0	0.84	26.5	70.5	15.7	10.1021/acsami.1c23513
PM6	Y6	BTP-MCA	-5.46	-3.6	-5.65	-4.08	-5.48	-3.58	14.29	0.87	26.74	73	17	10.1021/acsami.1c23513
PM6	Y6	BTP-MCA	-5.46	-3.6	-5.65	-4.08	-5.48	-3.58	20	0.89	26.69	69.9	16.6	10.1021/acsami.1c23513
PM6	Y6	BTP-MCA	-5.46	-3.6	-5.65	-4.08	-5.48	-3.58	33.33	0.89	26.64	69.3	16.4	10.1021/acsami.1c23513
PM6	Y6	BTP-MCA	-5.46	-3.6	-5.65	-4.08	-5.48	-3.58	50	0.88	22.03	68.5	13.3	10.1021/acsami.1c23513
PM6	Y6	BTP-MCA	-5.46	-3.6	-5.65	-4.08	-5.48	-3.58	100	0.8	0.12	30.1	0.03	10.1021/acsami.1c23513
PM6	ITCPTC	MeIC	-5.5	-3.61	-5.62	-3.96	-5.57	-3.92	0	0.96	17.149	0.744	12.236	10.1039/C8EE01700J
PM6	ITCPTC	MeIC	-5.5	-3.61	-5.62	-3.96	-5.57	-3.92	20	0.94	17.331	0.75	12.533	10.1039/C8EE01700J
PM6	ITCPTC	MeIC	-5.5	-3.61	-5.62	-3.96	-5.57	-3.92	40	0.971	18.068	0.769	13.481	10.1039/C8EE01700J
PM6	ITCPTC	MeIC	-5.5	-3.61	-5.62	-3.96	-5.57	-3.92	60	0.976	18.233	0.777	13.828	10.1039/C8EE01700J
PM6	ITCPTC	MeIC	-5.5	-3.61	-5.62	-3.96	-5.57	-3.92	80	0.981	18.169	0.755	13.456	10.1039/C8EE01700J
PM6	ITCPTC	MeIC	-5.5	-3.61	-5.62	-3.96	-5.57	-3.92	100	0.986	18.23	0.705	12.67	10.1039/C8EE01700J

PM6	BP4T-4F	BP3T-4F	-5.45	-3.65	-5.67	-3.89	-5.7	-3.82	0	0.851	25.43	78.06	16.89	10.1002/solr.202100365
PM6	BP4T-4F	BP3T-4F	-5.45	-3.65	-5.67	-3.89	-5.7	-3.82	20	0.855	25.27	78.09	16.87	10.1002/solr.202100365
PM6	BP4T-4F	BP3T-4F	-5.45	-3.65	-5.67	-3.89	-5.7	-3.82	40	0.862	25.07	78.24	16.91	10.1002/solr.202100365
PM6	BP4T-4F	BP3T-4F	-5.45	-3.65	-5.67	-3.89	-5.7	-3.82	60	0.865	24.51	77.56	16.44	10.1002/solr.202100365
PM6	BP4T-4F	BP3T-4F	-5.45	-3.65	-5.67	-3.89	-5.7	-3.82	100	0.872	23.09	76.34	15.37	10.1002/solr.202100365
PM6	Y6	TPIIC	-5.48	-3.6	-5.68	-4.06	-5.82	-3.92	0	0.84	26.2	72	15.8	10.1039/d2ta04463c
PM6	Y6	TPIIC	-5.48	-3.6	-5.68	-4.06	-5.82	-3.92	8.33	0.857	26.9	75.2	17.4	10.1039/d2ta04463c
PM6	Y6	TPIIC	-5.48	-3.6	-5.68	-4.06	-5.82	-3.92	12.5	0.861	27.5	74.4	17.7	10.1039/d2ta04463c
PM6	Y6	TPIIC	-5.48	-3.6	-5.68	-4.06	-5.82	-3.92	16.67	0.866	26.66	74.7	17.2	10.1039/d2ta04463c
PM6	Y6	TPIIC	-5.48	-3.6	-5.68	-4.06	-5.82	-3.92	20.83	0.864	26.3	72.4	16.4	10.1039/d2ta04463c
PM6	Y6	TPIIC	-5.48	-3.6	-5.68	-4.06	-5.82	-3.92	25	0.893	24.9	69.1	15.4	10.1039/d2ta04463c
PM6	Y6	TPIIC	-5.48	-3.6	-5.68	-4.06	-5.82	-3.92	100	0.931	16.3	68.8	10.5	10.1039/d2ta04463c
PM6	BTP-BO-4Cl	DTTC-4ClC9	-5.45	-3.65	-5.66	-4.09	-5.68	-4.02	0	0.85	26.54	75.86	17.11	10.1039/d2ta00716a
PM6	BTP-BO-4Cl	DTTC-4ClC9	-5.45	-3.65	-5.66	-4.09	-5.68	-4.02	15	0.864	27.12	77.76	18.21	10.1039/d2ta00716a
PM6	BTP-BO-4Cl	DTTC-4ClC9	-5.45	-3.65	-5.66	-4.09	-5.68	-4.02	25	0.871	26.75	76.4	17.8	10.1039/d2ta00716a
PM6	BTP-BO-4Cl	DTTC-4ClC9	-5.45	-3.65	-5.66	-4.09	-5.68	-4.02	50	0.891	24.06	75.27	16.14	10.1039/d2ta00716a
PM6	BTP-BO-4Cl	DTTC-4ClC9	-5.45	-3.65	-5.66	-4.09	-5.68	-4.02	75	0.905	23.09	71.77	14.99	10.1039/d2ta00716a
PM6	BTP-BO-4Cl	DTTC-4ClC9	-5.45	-3.65	-5.66	-4.09	-5.68	-4.02	100	0.945	21.05	72.92	14.5	10.1039/d2ta00716a
PM6	BTP-BO-4F	BTA1	-5.31	-3.42	-5.76	-4.35	-5.56	-3.62	0	0.848	25.72	76.51	16.73	10.1021/acsenergylett.2c01438
PM6	BTP-BO-4F	BTA1	-5.31	-3.42	-5.76	-4.35	-5.56	-3.62	10	0.872	25.69	76.42	17.12	10.1021/acsenergylett.2c01438
PM6	BTP-BO-4F	BTA1	-5.31	-3.42	-5.76	-4.35	-5.56	-3.62	20	0.873	25.12	75.58	16.64	10.1021/acsenergylett.2c01438
PM6	BTP-BO-4F	BTA1	-5.31	-3.42	-5.76	-4.35	-5.56	-3.62	30	0.867	24.87	73.79	15.91	10.1021/acsenergylett.2c01438
PM6	BTP-BO-4F	BTA1	-5.31	-3.42	-5.76	-4.35	-5.56	-3.62	50	0.866	20.66	64	11.45	10.1021/acsenergylett.2c01438
PM6	BTP-BO-4F	BTA2	-5.31	-3.42	-5.76	-4.35	-5.57	-3.53	10	0.869	26.76	78.55	18.27	10.1021/acsenergylett.2c01438
PM6	BTP-BO-4F	BTA2	-5.31	-3.42	-5.76	-4.35	-5.57	-3.53	20	0.878	25.56	76.32	17.13	10.1021/acsenergylett.2c01438
PM6	BTP-BO-4F	BTA2	-5.31	-3.42	-5.76	-4.35	-5.57	-3.53	30	0.885	25.02	74.59	16.52	10.1021/acsenergylett.2c01438
PM6	BTP-BO-4F	BTA2	-5.31	-3.42	-5.76	-4.35	-5.57	-3.53	50	0.893	22.91	67.47	13.8	10.1021/acsenergylett.2c01438
PM6	BTP-BO-4F	BTA3	-5.31	-3.42	-5.76	-4.35	-5.63	-3.82	10	0.863	26.46	77.87	17.78	10.1021/acsenergylett.2c01438
PM6	BTP-BO-4F	BTA3	-5.31	-3.42	-5.76	-4.35	-5.63	-3.82	20	0.863	25.41	76.13	16.69	10.1021/acsenergylett.2c01438
PM6	BTP-BO-4F	BTA3	-5.31	-3.42	-5.76	-4.35	-5.63	-3.82	30	0.866	24.9	74.75	16.12	10.1021/acsenergylett.2c01438
PM6	BTP-BO-4F	BTA3	-5.31	-3.42	-5.76	-4.35	-5.63	-3.82	50	0.874	22.4	69.71	13.65	10.1021/acsenergylett.2c01438
PM6	BTP-BO-4F	BTA3	-5.31	-3.42	-5.76	-4.35	-5.63	-3.82	100	1.189	7.86	65.17	6.09	10.1021/acsenergylett.2c01438
PM6	Y6	DFBT-TT6	-5.5	-3.56	-5.7	-4.1	-5.59	-3.65	0	0.845	25.2	74	15.76	10.1021/acs.chemmater.0c04297

PM6	Y6	DFBT-TT6	-5.5	-3.56	-5.7	-4.1	-5.59	-3.65	1	0.846	25.7	74	16.08	10.1021/acs.chemmater.0c04297
PM6	Y6	DFBT-TT6	-5.5	-3.56	-5.7	-4.1	-5.59	-3.65	3	0.845	26.56	76	17.05	10.1021/acs.chemmater.0c04297
PM6	Y6	DFBT-TT6	-5.5	-3.56	-5.7	-4.1	-5.59	-3.65	5	0.843	26.05	74	16.25	10.1021/acs.chemmater.0c04297
PM6	Y6	DFBT-TT6	-5.5	-3.56	-5.7	-4.1	-5.59	-3.65	7	0.844	24.82	70	14.66	10.1021/acs.chemmater.0c04297
PM6	Y6	DFBT-TT6	-5.5	-3.56	-5.7	-4.1	-5.59	-3.65	9	0.845	20.16	61	10.39	10.1021/acs.chemmater.0c04297
PM6	Y6	DFBT-DT6	-5.5	-3.56	-5.7	-4.1	-5.56	-3.6	1	0.844	25.5	74	15.71	10.1021/acs.chemmater.0c04297
PM6	Y6	DFBT-DT6	-5.5	-3.56	-5.7	-4.1	-5.56	-3.6	3	0.844	25.34	74	15.83	10.1021/acs.chemmater.0c04297
PM6	Y6	DFBT-DT6	-5.5	-3.56	-5.7	-4.1	-5.56	-3.6	5	0.843	25.51	72	15.48	10.1021/acs.chemmater.0c04297
PM6	Y6	DFBT-DT6	-5.5	-3.56	-5.7	-4.1	-5.56	-3.6	7	0.841	25.35	71	15.13	10.1021/acs.chemmater.0c04297
PM6	Y6	DFBT-DT6	-5.5	-3.56	-5.7	-4.1	-5.56	-3.6	9	0.841	24.51	69	15.22	10.1021/acs.chemmater.0c04297
PM6	CH-4Cl	F-2F	-5.46	-3.56	-5.74	-3.91	-5.78	-3.89	0	0.872	26.5	76.68	17.72	10.1039/D2EE01340A
PM6	CH-4Cl	F-2F	-5.46	-3.56	-5.74	-3.91	-5.78	-3.89	16.66	0.881	26.29	75.76	17.54	10.1039/D2EE01340A
PM6	CH-4Cl	F-2F	-5.46	-3.56	-5.74	-3.91	-5.78	-3.89	25	0.896	26.69	76.17	18.22	10.1039/D2EE01340A
D18-Cl	Y6	m-BTP-PhC6	-5.26	-3.39	-5.63	-3.96	-5.45	-3.78	0	0.86	26.33	76.66	17.37	10.1002/solr.202200508
D18-Cl	Y6	m-BTP-PhC6	-5.26	-3.39	-5.63	-3.96	-5.45	-3.78	10	0.865	26.69	77.21	17.84	10.1002/solr.202200508
D18-Cl	Y6	m-BTP-PhC6	-5.26	-3.39	-5.63	-3.96	-5.45	-3.78	20	0.871	26.8	77.32	18.03	10.1002/solr.202200508
D18-Cl	Y6	m-BTP-PhC6	-5.26	-3.39	-5.63	-3.96	-5.45	-3.78	50	0.876	25.96	77.03	17.48	10.1002/solr.202200508
D18-Cl	Y6	m-BTP-PhC6	-5.26	-3.39	-5.63	-3.96	-5.45	-3.78	100	0.89	24.69	77.08	16.94	10.1002/solr.202200508
PM6	Y7	BTA-UD-4F	-5.45	-3.64	-5.65	-4.02	-5.4	-3.91	0	0.82	25.56	69.89	15.1	10.1016/j.cej.2022.137621
PM6	Y7	BTA-UD-4F	-5.45	-3.64	-5.65	-4.02	-5.4	-3.91	6.66	0.8	26.86	71.12	15.86	10.1016/j.cej.2022.137621
PM6	Y7	BTA-UD-4F	-5.45	-3.64	-5.65	-4.02	-5.4	-3.91	13.33	0.83	26.76	72.35	16.18	10.1016/j.cej.2022.137621
PM6	Y7	BTA-UD-4F	-5.45	-3.64	-5.65	-4.02	-5.4	-3.91	20	0.8	26.21	71.35	15.32	10.1016/j.cej.2022.137621
PM6	Y7	BTA-UD-4F	-5.45	-3.64	-5.65	-4.02	-5.4	-3.91	33.33	0.78	25.3	67.29	13.4	10.1016/j.cej.2022.137621
PM6	Y7	BTA-UD-4F	-5.45	-3.64	-5.65	-4.02	-5.4	-3.91	100	0.81	24.98	67.98	14.32	10.1016/j.cej.2022.137621
PM6	L8-BO	BTP-S10	-5.48	-3.64	-5.72	-3.9	-5.57	-3.85	0	0.883	25.98	79	18.3	10.1002/aenm.202201076
PM6	L8-BO	BTP-S10	-5.48	-3.64	-5.72	-3.9	-5.57	-3.85	15	0.892	26.24	79.69	18.69	10.1002/aenm.202201076
PM6	L8-BO	BTP-S10	-5.48	-3.64	-5.72	-3.9	-5.57	-3.85	20	0.898	26.8	80.22	19.26	10.1002/aenm.202201076
PM6	L8-BO	BTP-S10	-5.48	-3.64	-5.72	-3.9	-5.57	-3.85	25	0.908	26.11	79.63	18.83	10.1002/aenm.202201076
PM6	L8-BO	BTP-S10	-5.48	-3.64	-5.72	-3.9	-5.57	-3.85	100	0.943	20.54	69.46	13.44	10.1002/aenm.202201076
D18	Y6	BTIC-γ-2CN	-5.51	-2.77	-5.65	-4.1	-5.73	-3.92	0	0.867	25.29	77.08	16.9	10.1039/d2tc03701g
D18	Y6	BTIC-γ-2CN	-5.51	-2.77	-5.65	-4.1	-5.73	-3.92	5	0.879	25.15	78.07	17.26	10.1039/d2tc03701g
D18	Y6	BTIC-γ-2CN	-5.51	-2.77	-5.65	-4.1	-5.73	-3.92	10	0.882	25.27	79.14	17.61	10.1039/d2tc03701g
D18	Y6	BTIC-γ-2CN	-5.51	-2.77	-5.65	-4.1	-5.73	-3.92	20	0.883	25.38	77.22	17.3	10.1039/d2tc03701g

D18	Y6	BTIC-γ-2CN	-5.51	-2.77	-5.65	-4.1	-5.73	-3.92	30	0.893	24.74	76.84	17.22	10.1039/d2tc03701g
D18	Y6	BTIC-γ-2CN	-5.51	-2.77	-5.65	-4.1	-5.73	-3.92	100	0.926	22.54	76.76	16.03	10.1039/d2tc03701g
PTB7-Th	IEICO-4F	FLCPDT	-5.24	-3.64	-5.44	-4.19	-5.38	-3.72	0	0.7	26.08	63.56	11.67	10.1002/solr.202200302
PTB7-Th	IEICO-4F	FLCPDT	-5.24	-3.64	-5.44	-4.19	-5.38	-3.72	10	0.71	26.1	65.67	12.18	10.1002/solr.202200302
PTB7-Th	IEICO-4F	FLCPDT	-5.24	-3.64	-5.44	-4.19	-5.38	-3.72	20	0.73	26.3	66.51	12.77	10.1002/solr.202200302
PTB7-Th	IEICO-4F	FLCPDT	-5.24	-3.64	-5.44	-4.19	-5.38	-3.72	30	0.75	25.56	56.61	10.66	10.1002/solr.202200302
PTB7-Th	IEICO-4F	FLCPDT	-5.24	-3.64	-5.44	-4.19	-5.38	-3.72	50	0.76	20.43	47.21	7.3	10.1002/solr.202200302
PTB7-Th	IEICO-4F	FLCPDT	-5.24	-3.64	-5.44	-4.19	-5.38	-3.72	100	0.89	9.77	40.02	3.48	10.1002/solr.202200302
PTB7-Th	IEICO-4F	SFCPDT	-5.24	-3.64	-5.44	-4.19	-5.46	-3.79	10	0.73	27.56	66.38	13.29	10.1002/solr.202200302
PTB7-Th	IEICO-4F	SFCPDT	-5.24	-3.64	-5.44	-4.19	-5.46	-3.79	20	0.74	25.59	56.73	10.71	10.1002/solr.202200302
PTB7-Th	IEICO-4F	SFCPDT	-5.24	-3.64	-5.44	-4.19	-5.46	-3.79	30	0.75	23.43	49.67	8.63	10.1002/solr.202200302
PTB7-Th	IEICO-4F	SFCPDT	-5.24	-3.64	-5.44	-4.19	-5.46	-3.79	50	0.76	19.12	43.04	6.24	10.1002/solr.202200302
PTB7-Th	IEICO-4F	SFCPDT	-5.24	-3.64	-5.44	-4.19	-5.46	-3.79	100	0.86	9.88	44.24	3.76	10.1002/solr.202200302
PM6	C8C8-4Cl	BDC-4F-C8	-5.5	-3.66	-5.7	-4.3	-5.66	-4.26	0	0.818	24.01	71.1	13.96	10.1016/j.nanoen.2022.107186
PM6	C8C8-4Cl	BDC-4F-C8	-5.5	-3.66	-5.7	-4.3	-5.66	-4.26	8.33	0.821	24.27	72.56	14.45	10.1016/j.nanoen.2022.107186
PM6	C8C8-4Cl	BDC-4F-C8	-5.5	-3.66	-5.7	-4.3	-5.66	-4.26	16.66	0.833	24.56	73.41	15.01	10.1016/j.nanoen.2022.107186
PM6	C8C8-4Cl	BDC-4F-C8	-5.5	-3.66	-5.7	-4.3	-5.66	-4.26	25	0.839	24.33	71.03	14.49	10.1016/j.nanoen.2022.107186
PM6	C8C8-4Cl	BDC-4F-C8	-5.5	-3.66	-5.7	-4.3	-5.66	-4.26	100	0.895	21.4	63.42	12.14	10.1016/j.nanoen.2022.107186
PTB7-Th	IEICO-4F	P-	-5.24	-3.62	-5.44	-4.19	-5.18	-3.5	10	0.713	20.66	70.52	10.38	10.1016/j.nanoen.2022.107186
PTB7-Th	IEICO-4F	P-	-5.24	-3.62	-5.44	-4.19	-5.18	-3.5	15	0.715	21.5	70.78	10.88	10.1016/j.nanoen.2022.107186
PTB7-Th	IEICO-4F	P-	-5.24	-3.62	-5.44	-4.19	-5.18	-3.5	20	0.711	20.48	72.81	10.6	10.1016/j.nanoen.2022.107186
PTB7-Th	IEICO-4F	P-	-5.24	-3.62	-5.44	-4.19	-5.18	-3.5	25	0.712	19.61	72.22	10.08	10.1016/j.nanoen.2022.107186
PTB7-Th	IEICO-4F	P-	-5.24	-3.62	-5.44	-4.19	-5.18	-3.5	30	0.715	18.5	71.86	9.51	10.1016/j.nanoen.2022.107186
PTB7-Th	IEICO-4F	P-	-5.24	-3.62	-5.44	-4.19	-5.18	-3.5	100	0.622	3.35	31.45	0.66	10.1016/j.nanoen.2022.107186
PM6	L8-BO	IT-M	-5.43	-3.56	-5.68	-3.9	-5.6	-3.71	0	0.871	25.77	78.68	17.66	10.1039/d2ee01894b
PM6	L8-BO	IT-M	-5.43	-3.56	-5.68	-3.9	-5.6	-3.71	2.5	0.873	26.08	79.15	18.02	10.1039/d2ee01894b
PM6	L8-BO	IT-M	-5.43	-3.56	-5.68	-3.9	-5.6	-3.71	4.17	0.875	26.22	79.29	18.19	10.1039/d2ee01894b
PM6	L8-BO	IT-M	-5.43	-3.56	-5.68	-3.9	-5.6	-3.71	5.83	0.867	25.89	78.41	17.6	10.1039/d2ee01894b
PM6	N3	IT-M	-5.43	-3.56	-5.7	-3.88	-5.6	-3.71	0	0.831	25.49	77.1	16.33	10.1039/d2ee01894b
PM6	N3	IT-M	-5.43	-3.56	-5.7	-3.88	-5.6	-3.71	2.5	0.853	25.84	77.15	17.01	10.1039/d2ee01894b
PM6	N3	IT-M	-5.43	-3.56	-5.7	-3.88	-5.6	-3.71	4.17	0.856	25.96	77.45	17.21	10.1039/d2ee01894b
PM6	N3	IT-M	-5.43	-3.56	-5.7	-3.88	-5.6	-3.71	5.83	0.85	25.6	75.89	16.51	10.1039/d2ee01894b
PM6	BTP-eC9	IT-M	-5.43	-3.56	-5.59	-3.98	-5.6	-3.71	0	0.847	26.61	77.26	17.41	10.1039/d2ee01894b

PM6	BTP-eC9	IT-M	-5.43	-3.56	-5.59	-3.98	-5.6	-3.71	2.5	0.859	26.65	77.58	17.76	10.1039/d2ee01894b
PM6	BTP-eC9	IT-M	-5.43	-3.56	-5.59	-3.98	-5.6	-3.71	4.17	0.865	26.69	78.18	18.05	10.1039/d2ee01894b
PM6	BTP-eC9	IT-M	-5.43	-3.56	-5.59	-3.98	-5.6	-3.71	5.83	0.852	26.52	76.13	17.2	10.1039/d2ee01894b
PM6	PY-IT	N2200	-5.43	-3.56	-5.65	-3.79	-5.8	-3.8	0	0.941	22.89	71.25	15.35	10.1039/d2ee01894b
PM6	PY-IT	N2200	-5.43	-3.56	-5.65	-3.79	-5.8	-3.8	1	0.942	23.01	72.29	15.67	10.1039/d2ee01894b
PM6	PY-IT	N2200	-5.43	-3.56	-5.65	-3.79	-5.8	-3.8	3	0.943	23.05	73.76	16.03	10.1039/d2ee01894b
PM6	PY-IT	N2200	-5.43	-3.56	-5.65	-3.79	-5.8	-3.8	5	0.94	22.86	71.15	15.29	10.1039/d2ee01894b
PTB7-Th	SF-4PDI	TPE-4PDI	-5.38	-3.78	-5.92	-4.01	-6.29	-4.2	0	0.861	14.46	42.8	5.33	10.1016/j.dyepig.2017.02.043
PTB7-Th	SF-4PDI	TPE-4PDI	-5.38	-3.78	-5.92	-4.01	-6.29	-4.2	5	0.86	15.21	44.5	5.78	10.1016/j.dyepig.2017.02.043
PTB7-Th	SF-4PDI	TPE-4PDI	-5.38	-3.78	-5.92	-4.01	-6.29	-4.2	10	0.859	14.94	48.2	6.19	10.1016/j.dyepig.2017.02.043
PTB7-Th	SF-4PDI	TPE-4PDI	-5.38	-3.78	-5.92	-4.01	-6.29	-4.2	15	0.846	13.08	38.2	4.12	10.1016/j.dyepig.2017.02.043
PTB7-Th	SF-4PDI	TPE-4PDI	-5.38	-3.78	-5.92	-4.01	-6.29	-4.2	20	0.829	11.72	38	3.73	10.1016/j.dyepig.2017.02.043
PTB7-Th	SF-4PDI	TPE-4PDI	-5.38	-3.78	-5.92	-4.01	-6.29	-4.2	100	0.716	4.44	34.8	1.04	10.1016/j.dyepig.2017.02.043
PBT1-C	MeIC	MeIC2	-5.43	-3.36	-5.47	-3.82	-5.56	-3.97	0	0.942	16.54	73.6	11.47	10.1002/smll.201802983
PBT1-C	MeIC	MeIC2	-5.43	-3.36	-5.47	-3.82	-5.56	-3.97	10	0.94	17.01	74.9	11.97	10.1002/smll.201802983
PBT1-C	MeIC	MeIC2	-5.43	-3.36	-5.47	-3.82	-5.56	-3.97	20	0.938	17.41	75	12.24	10.1002/smll.201802983
PBT1-C	MeIC	MeIC2	-5.43	-3.36	-5.47	-3.82	-5.56	-3.97	30	0.936	17.84	75.2	12.55	10.1002/smll.201802983
PBT1-C	MeIC	MeIC2	-5.43	-3.36	-5.47	-3.82	-5.56	-3.97	40	0.934	17.67	74.9	12.37	10.1002/smll.201802983
PBT1-C	MeIC	MeIC2	-5.43	-3.36	-5.47	-3.82	-5.56	-3.97	60	0.931	17.55	73.9	12.08	10.1002/smll.201802983
PBT1-C	MeIC	MeIC2	-5.43	-3.36	-5.47	-3.82	-5.56	-3.97	80	0.927	17.38	73.1	11.77	10.1002/smll.201802983
PBT1-C	MeIC	MeIC2	-5.43	-3.36	-5.47	-3.82	-5.56	-3.97	100	0.923	17.14	72.1	11.41	10.1002/smll.201802983
PBDB-T	IT-M	PDCBT	-5.28	-3.48	-5.51	-3.91	-5.26	-3	0	0.933	17.16	63.5	10.17	10.2494/photopolymer.31.177
PBDB-T	IT-M	PDCBT	-5.28	-3.48	-5.51	-3.91	-5.26	-3	5	0.929	17.73	66.4	10.94	10.2494/photopolymer.31.177
PBDB-T	IT-M	PDCBT	-5.28	-3.48	-5.51	-3.91	-5.26	-3	10	0.93	17.97	66.7	11.2	10.2494/photopolymer.31.177
PBDB-T	IT-M	PDCBT	-5.28	-3.48	-5.51	-3.91	-5.26	-3	15	0.927	16.81	59.5	9.27	10.2494/photopolymer.31.177
PBDB-T	IDT6CN-M	ITCPTC	-5.21	-3.41	-5.64	-3.92	-5.64	-3.98	0	0.915	16	75.3	11.02	10.1063/5.0022887
PBDB-T	IDT6CN-M	ITCPTC	-5.21	-3.41	-5.64	-3.92	-5.64	-3.98	10	0.91	16.21	75.4	11.12	10.1063/5.0022887
PBDB-T	IDT6CN-M	ITCPTC	-5.21	-3.41	-5.64	-3.92	-5.64	-3.98	20	0.904	16.44	75.4	11.21	10.1063/5.0022887
PBDB-T	IDT6CN-M	ITCPTC	-5.21	-3.41	-5.64	-3.92	-5.64	-3.98	30	0.891	16.67	75.6	11.23	10.1063/5.0022887
PBDB-T	IDT6CN-M	ITCPTC	-5.21	-3.41	-5.64	-3.92	-5.64	-3.98	40	0.886	16.94	75.8	11.38	10.1063/5.0022887
PBDB-T	IDT6CN-M	ITCPTC	-5.21	-3.41	-5.64	-3.92	-5.64	-3.98	50	0.878	17.25	76.2	11.54	10.1063/5.0022887
PBDB-T	IDT6CN-M	ITCPTC	-5.21	-3.41	-5.64	-3.92	-5.64	-3.98	60	0.875	17.81	76.5	11.92	10.1063/5.0022887
PBDB-T	IDT6CN-M	ITCPTC	-5.21	-3.41	-5.64	-3.92	-5.64	-3.98	70	0.867	17.66	75.4	11.54	10.1063/5.0022887

PBDB-T	IDT6CN-M	ITCPTC	-5.21	-3.41	-5.64	-3.92	-5.64	-3.98	80	0.853	17.62	74.7	11.23	10.1063/5.0022887
PBDB-T	IDT6CN-M	ITCPTC	-5.21	-3.41	-5.64	-3.92	-5.64	-3.98	90	0.842	17.51	73.3	10.81	10.1063/5.0022887
PBDB-T	IDT6CN-M	ITCPTC	-5.21	-3.41	-5.64	-3.92	-5.64	-3.98	100	0.836	17.44	72.1	10.51	10.1063/5.0022887
PBDB-T	ITIC-Th	TPE-4PDI	-5.53	-3.53	-5.66	-3.98	-5.93	-3.88	0	0.85	16.45	69.7	9.75	10.1039/c7ta06237k
PBDB-T	ITIC-Th	TPE-4PDI	-5.53	-3.53	-5.66	-3.98	-5.93	-3.88	10	0.87	17.2	72.6	10.82	10.1039/c7ta06237k
PBDB-T	ITIC-Th	TPE-4PDI	-5.53	-3.53	-5.66	-3.98	-5.93	-3.88	25	0.89	15.24	70.3	9.52	10.1039/c7ta06237k
PBDB-T	ITIC-Th	TPE-4PDI	-5.53	-3.53	-5.66	-3.98	-5.93	-3.88	50	0.91	13.53	65.6	8.08	10.1039/c7ta06237k
PBDB-T	ITIC-Th	TPE-4PDI	-5.53	-3.53	-5.66	-3.98	-5.93	-3.88	100	0.95	8.92	55.8	4.73	10.1039/c7ta06237k
PBDB-T	ITIC	P1	-5.21	-3.41	-5.61	-4.02	-5.39	-3.5	0	0.9	16.64	71.24	10.66	10.1039/c8ee01564c
PBDB-T	ITIC	P1	-5.21	-3.41	-5.61	-4.02	-5.39	-3.5	5	0.9	17.98	77.33	12.51	10.1039/c8ee01564c
PBDB-T	ITIC	P1	-5.21	-3.41	-5.61	-4.02	-5.39	-3.5	10	0.91	17.48	75.78	11.92	10.1039/c8ee01564c
PBDB-T	ITIC	P1	-5.21	-3.41	-5.61	-4.02	-5.39	-3.5	100	0.94	8.15	54.44	3.85	10.1039/c8ee01564c
PBDB-T	ITIC	IDT-T	-5.21	-3.41	-5.47	-3.76	-5.73	-3.59	0	0.9	16.8	69.3	10.5	10.1016/j.joule.2018.08.002
PBDB-T	ITIC	IDT-T	-5.21	-3.41	-5.47	-3.76	-5.73	-3.59	1	0.92	17.3	70.5	11.2	10.1016/j.joule.2018.08.002
PBDB-T	ITIC	IDT-T	-5.21	-3.41	-5.47	-3.76	-5.73	-3.59	20	0.935	17.9	73.1	12.2	10.1016/j.joule.2018.08.002
PBDB-T	ITIC	IDT-T	-5.21	-3.41	-5.47	-3.76	-5.73	-3.59	30	0.945	16.7	72	11.4	10.1016/j.joule.2018.08.002
PBDB-T	ITIC	IDT-T	-5.21	-3.41	-5.47	-3.76	-5.73	-3.59	50	0.955	15.3	67.2	9.82	10.1016/j.joule.2018.08.002
PBDB-T	ITIC	IDT-T	-5.21	-3.41	-5.47	-3.76	-5.73	-3.59	80	0.975	13	61	7.74	10.1016/j.joule.2018.08.002
PBDB-T	ITIC	IDT-T	-5.21	-3.41	-5.47	-3.76	-5.73	-3.59	100	1.05	11.8	59.8	7.38	10.1016/j.joule.2018.08.002
J71	ITIC	MeIC2	-5.4	-3.24	-5.51	-3.92	-5.56	-3.97	0	0.93	16.4	70	10.7	10.1039/C8TA03453B
J71	ITIC	MeIC2	-5.4	-3.24	-5.51	-3.92	-5.56	-3.97	10	0.92	17.4	70.2	11.2	10.1039/C8TA03453B
J71	ITIC	MeIC2	-5.4	-3.24	-5.51	-3.92	-5.56	-3.97	20	0.91	18.1	70.5	11.6	10.1039/C8TA03453B
J71	ITIC	MeIC2	-5.4	-3.24	-5.51	-3.92	-5.56	-3.97	30	0.9	17.8	70.1	11.2	10.1039/C8TA03453B
J71	ITIC	MeIC2	-5.4	-3.24	-5.51	-3.92	-5.56	-3.97	50	0.898	17.7	69.6	11.1	10.1039/C8TA03453B
J71	ITIC	MeIC2	-5.4	-3.24	-5.51	-3.92	-5.56	-3.97	100	0.885	16.9	69.4	10.2	10.1039/C8TA03453B
PBDB-TF	IT-2F	IT-4F	-5.56	-3.5	-5.64	-4.03	-5.69	-4.07	0	0.93	19.32	70	12.4	10.1039/c8ta09830a
PBDB-TF	IT-2F	IT-4F	-5.56	-3.5	-5.64	-4.03	-5.69	-4.07	20	0.91	20.4	72.9	13.29	10.1039/c8ta09830a
PBDB-TF	IT-2F	IT-4F	-5.56	-3.5	-5.64	-4.03	-5.69	-4.07	40	0.9	20.35	74.2	13.58	10.1039/c8ta09830a
PBDB-TF	IT-2F	IT-4F	-5.56	-3.5	-5.64	-4.03	-5.69	-4.07	50	0.89	20.67	75.5	13.89	10.1039/c8ta09830a
PBDB-TF	IT-2F	IT-4F	-5.56	-3.5	-5.64	-4.03	-5.69	-4.07	60	0.88	20.83	76	13.93	10.1039/c8ta09830a
PBDB-TF	IT-2F	IT-4F	-5.56	-3.5	-5.64	-4.03	-5.69	-4.07	80	0.88	20.7	77.1	14.04	10.1039/c8ta09830a
PBDB-TF	IT-2F	IT-4F	-5.56	-3.5	-5.64	-4.03	-5.69	-4.07	100	0.86	20.82	73.9	13.33	10.1039/c8ta09830a
PTFB-O	ITIC-Th	IEIC-Th	-5.37	-3.36	-5.58	-3.94	-5.46	-3.86	0	0.92	16.8	66	10.2	10.1002/aenm.201701370

PTFB-O	ITIC-Th	IEIC-Th	-5.37	-3.36	-5.58	-3.94	-5.46	-3.86	20	0.933	16.6	68	10.5	10.1002/aenm.201701370
PTFB-O	ITIC-Th	IEIC-Th	-5.37	-3.36	-5.58	-3.94	-5.46	-3.86	40	0.948	16.4	72	11.2	10.1002/aenm.201701370
PTFB-O	ITIC-Th	IEIC-Th	-5.37	-3.36	-5.58	-3.94	-5.46	-3.86	50	0.968	15	70	10.2	10.1002/aenm.201701370
PTFB-O	ITIC-Th	IEIC-Th	-5.37	-3.36	-5.58	-3.94	-5.46	-3.86	80	0.988	14.8	68	9.9	10.1002/aenm.201701370
PTFB-O	ITIC-Th	IEIC-Th	-5.37	-3.36	-5.58	-3.94	-5.46	-3.86	100	1.018	13	66	8.7	10.1002/aenm.201701370
PTFB-O	ITIC-Th	SF-PDI2	-5.37	-3.36	-5.58	-3.94	-5.82	-3.8	20	0.95	15.1	73	10.5	10.1002/aenm.201701370
PTFB-O	ITIC-Th	SF-PDI2	-5.37	-3.36	-5.58	-3.94	-5.82	-3.8	40	0.957	13.6	73	9.5	10.1002/aenm.201701370
PTFB-O	ITIC-Th	SF-PDI2	-5.37	-3.36	-5.58	-3.94	-5.82	-3.8	50	0.965	12.6	64	7.8	10.1002/aenm.201701370
PTFB-O	ITIC-Th	SF-PDI2	-5.37	-3.36	-5.58	-3.94	-5.82	-3.8	80	0.978	9.8	63	6	10.1002/aenm.201701370
PTFB-O	ITIC-Th	SF-PDI2	-5.37	-3.36	-5.58	-3.94	-5.82	-3.8	100	0.986	8.7	55	4.7	10.1002/aenm.201701370
PTFB-O	ITIC-Th	TPE-PDI4	-5.37	-3.36	-5.58	-3.94	-5.82	-3.77	20	0.942	14.7	71	9.8	10.1002/aenm.201701370
PTFB-O	ITIC-Th	TPE-PDI4	-5.37	-3.36	-5.58	-3.94	-5.82	-3.77	40	0.951	12.5	71	8.4	10.1002/aenm.201701370
PTFB-O	ITIC-Th	TPE-PDI4	-5.37	-3.36	-5.58	-3.94	-5.82	-3.77	50	0.958	10.6	65	6.6	10.1002/aenm.201701370
PTFB-O	ITIC-Th	TPE-PDI4	-5.37	-3.36	-5.58	-3.94	-5.82	-3.77	80	0.965	9.2	57	5	10.1002/aenm.201701370
PTFB-O	ITIC-Th	TPE-PDI4	-5.37	-3.36	-5.58	-3.94	-5.82	-3.77	100	0.976	7	60	4.1	10.1002/aenm.201701370
PSTZ	ITIC	IDIC	-5.44	-3.48	-5.6	-3.85	-5.67	-3.91	0	1.01	15.4	52.3	8.13	10.1016/j.nanoen.2017.05.060
PSTZ	ITIC	IDIC	-5.44	-3.48	-5.6	-3.85	-5.67	-3.91	30	0.974	16.3	52.8	8.38	10.1016/j.nanoen.2017.05.060
PSTZ	ITIC	IDIC	-5.44	-3.48	-5.6	-3.85	-5.67	-3.91	50	0.971	16.5	54.1	8.67	10.1016/j.nanoen.2017.05.060
PSTZ	ITIC	IDIC	-5.44	-3.48	-5.6	-3.85	-5.67	-3.91	80	0.962	16.3	60.8	9.53	10.1016/j.nanoen.2017.05.060
PSTZ	ITIC	IDIC	-5.44	-3.48	-5.6	-3.85	-5.67	-3.91	90	0.947	16.5	65.4	10.2	10.1016/j.nanoen.2017.05.060
PSTZ	ITIC	IDIC	-5.44	-3.48	-5.6	-3.85	-5.67	-3.91	95	0.931	15.6	64.1	9.31	10.1016/j.nanoen.2017.05.060
PSTZ	ITIC	IDIC	-5.44	-3.48	-5.6	-3.85	-5.67	-3.91	100	0.928	14.7	59.1	8.06	10.1016/j.nanoen.2017.05.060
PCE-10	BT-CIC	TT-FIC	-5.22	-3.21	-5.49	-4.09	-5.42	-4.13	0	0.695	22.3	70	10.8	10.1002/adma.201804416
PCE-10	BT-CIC	TT-FIC	-5.22	-3.21	-5.49	-4.09	-5.42	-4.13	16.67	0.696	23.8	71	11.7	10.1002/adma.201804416
PCE-10	BT-CIC	TT-FIC	-5.22	-3.21	-5.49	-4.09	-5.42	-4.13	28.57	0.693	25.5	71	12.6	10.1002/adma.201804416
PCE-10	BT-CIC	TT-FIC	-5.22	-3.21	-5.49	-4.09	-5.42	-4.13	37.5	0.687	26.6	66	12.1	10.1002/adma.201804416
PCE-10	BT-CIC	TT-FIC	-5.22	-3.21	-5.49	-4.09	-5.42	-4.13	100	0.65	24.7	67	10.8	10.1002/adma.201804416
PDTF-TZNT	IT-M	meta-TrBRCN	-5.16	-3.1	-5.58	-3.98	-5.95	-3.73	0	0.75	10.15	58.1	4.42	10.1002/adma.201804421
PDTF-TZNT	IT-M	meta-TrBRCN	-5.16	-3.1	-5.58	-3.98	-5.95	-3.73	9.09	0.81	17.78	73.6	10.6	10.1002/adma.201804421
PDTF-TZNT	IT-M	meta-TrBRCN	-5.16	-3.1	-5.58	-3.98	-5.95	-3.73	15.38	0.82	18.54	75.5	11.48	10.1002/adma.201804421
PDTF-TZNT	IT-M	meta-TrBRCN	-5.16	-3.1	-5.58	-3.98	-5.95	-3.73	21.43	0.83	18.01	71.3	10.66	10.1002/adma.201804421
PDTF-TZNT	IT-M	meta-TrBRCN	-5.16	-3.1	-5.58	-3.98	-5.95	-3.73	26.67	0.84	17.39	68.2	9.96	10.1002/adma.201804421
PTB7-Th	COi8DFIC	BDTThIT-4F	-5.23	-3.52	-5.46	-3.9	-5.57	-3.92	0	0.71	23.45	68.9	11.47	10.1002/aenm.201801968

PTB7-Th	COi8DFIC	BDTThIT-4F	-5.23	-3.52	-5.46	-3.9	-5.57	-3.92	10	0.714	23.88	71.2	12.14	10.1002/aenm.201801968
PTB7-Th	COi8DFIC	BDTThIT-4F	-5.23	-3.52	-5.46	-3.9	-5.57	-3.92	20	0.721	24.88	72.9	13.08	10.1002/aenm.201801968
PTB7-Th	COi8DFIC	BDTThIT-4F	-5.23	-3.52	-5.46	-3.9	-5.57	-3.92	30	0.725	24.29	72.5	12.77	10.1002/aenm.201801968
PTB7-Th	COi8DFIC	BDTThIT-4F	-5.23	-3.52	-5.46	-3.9	-5.57	-3.92	50	0.731	21.76	68.9	10.96	10.1002/aenm.201801968
PTB7-Th	COi8DFIC	BDTThIT-4F	-5.23	-3.52	-5.46	-3.9	-5.57	-3.92	100	0.746	17.25	70.8	9.25	10.1002/aenm.201801968

References

1. Wang, L.; Zhu, Z.; Yang, S.; Fan, J.; Huang, S.; Yang, S.; Li, H.; Liu, H. Boosting the performance of all-polymer solar cells via incorporating a versatile small-molecule non-fullerene acceptor. *Synth. Met.* **2023**, *293*, https://doi.org/10.1016/j.synthmet.2023.117292.
2. Liu, X.; Liu, Y.; Ni, Y.; Fu, P.; Wang, X.; Yang, Q.; Guo, X.; Li, C. Reducing non-radiative recombination energy loss via a fluorescence intensifier for efficient and stable ternary organic solar cells. *Mater. Horizons* **2021**, *8*, 2335–2342, https://doi.org/10.1039/d1mh00868d.
3. Lan, A.; Lv, Y.; Zhu, J.; Lu, H.; Do, H.; Chen, Z.-K.; Zhou, J.; Wang, H.; Chen, F.; Zhou, E. High-performance ternary organic solar cells through incorporation of a series of A2-A1-D-A1-A2 type nonfullerene acceptors with different terminal groups. *ACS Energy Lett.* **2022**, *7*, 2845–2855.
4. Huang, T.; Zhang, Z.; Wang, D.; Zhang, Y.; Deng, Z.; Huang, Y.; Liao, Q.; Zhang, J. 18.7% Efficiency Ternary Organic Solar Cells Using Two Non-Fullerene Acceptors with Excellent Compatibility. *ACS Appl. Energy Mater.* **2023**, *6*, 3126–3134, https://doi.org/10.1021/acsaem.3c00093.
5. Chen, T.; Li, S.; Li, Y.; Chen, Z.; Wu, H.; Lin, Y.; Gao, Y.; Wang, M.; Ding, G.; Min, J.; et al. Compromising Charge Generation and Recombination of Organic Photovoltaics with Mixed Diluent Strategy for Certified 19.4% Efficiency. *Adv. Mater.* **2023**, *35*, e2300400, https://doi.org/10.1002/adma.202300400.
